# How 5000 independent rowers coordinate their strokes in order to row into the sunlight: Phototaxis in the multicellular green alga *Volvox*

**DOI:** 10.1186/1741-7007-8-103

**Published:** 2010-07-27

**Authors:** Noriko Ueki, Shigeru Matsunaga, Isao Inouye, Armin Hallmann

**Affiliations:** 1 Department of Cellular and Developmental Biology of Plants, University of Bielefeld, Universitätsstr. 25, 33615 Bielefeld, Germany; 2 Institute of Biological Sciences, University of Tsukuba, Tsukuba, Ibaraki, 305-8577 Japan; 3 The Promotion Center for Research and Education, Graduate University for Advanced Studies (Sokendai), Hayama, Kanagawa, 240-0193 Japan

## Abstract

**Background:**

The evolution of multicellular motile organisms from unicellular ancestors required the utilization of previously evolved tactic behavior in a multicellular context. Volvocine green algae are uniquely suited for studying tactic responses during the transition to multicellularity because they range in complexity from unicellular to multicellular genera. Phototactic responses are essential for these flagellates because they need to orientate themselves to receive sufficient light for photosynthesis, but how does a multicellular organism accomplish phototaxis without any known direct communication among cells? Several aspects of the photoresponse have previously been analyzed in volvocine algae, particularly in the unicellular alga *Chlamydomonas*.

**Results:**

In this study, the phototactic behavior in the spheroidal, multicellular volvocine green alga *Volvox rousseletii *(Volvocales, Chlorophyta) was analyzed. In response to light stimuli, not only did the flagella waveform and beat frequency change, but the effective stroke was reversed. Moreover, there was a photoresponse gradient from the anterior to the posterior pole of the spheroid, and only cells of the anterior hemisphere showed an effective response. The latter caused a reverse of the fluid flow that was confined to the anterior hemisphere. The responsiveness to light is consistent with an anterior-to-posterior size gradient of eyespots. At the posterior pole, the eyespots are tiny or absent, making the corresponding cells appear to be blind. Pulsed light stimulation of an immobilized spheroid was used to simulate the light fluctuation experienced by a rotating spheroid during phototaxis. The results demonstrated that in free-swimming spheroids, only those cells of the anterior hemisphere that face toward the light source reverse the beating direction in the presence of illumination; this behavior results in phototactic turning. Moreover, positive phototaxis is facilitated by gravitational forces. Under our conditions, *V. rousseletii *spheroids showed no negative phototaxis.

**Conclusions:**

On the basis of our results, we developed a mechanistic model that predicts the phototactic behavior in *V. rousseletii*. The model involves photoresponses, periodically changing light conditions, morphological polarity, rotation of the spheroid, two modes of flagellar beating, and the impact of gravity. Our results also indicate how recently evolved multicellular organisms adapted the phototactic capabilities of their unicellular ancestors to multicellular life.

## Background

The ability of motile organisms to sense changes in external parameters is crucial in finding the best environmental conditions. These tactic responses were already developed in early single-celled life. When multicellular motile organisms evolved from unicellular life, previous achievements like tactic behavior had to be modified to fulfill the requirements of multicellularity [1-4]. The volvocine green algae, a group of species closely related to the "genus" *Volvox *within the order Volvocales (Chlorophyta), represent an excellent set of organisms for analyzing (photo)tactic responses during evolution of multicellularity [4-6]. This group of algae spans the full range of complexity from unicellular genera, such as *Chlamydomonas*, to colonial genera, such as *Eudorina*, to multicellular genera, such as *Volvox *[5].

Phylogenetic analyses indicate that multicellularity evolved much more recently in volvocine green algae than in any other group [7]. Phylogenetic analyses of the order Volvocales also revealed a polyphyletic origin of the "genus" *Volvox *[5,8-13]. A few species of this genus, including *Volvox rousseletii*, form a small but robust monophyletic group, which is called the section *Volvox *[10,12,14-16]. All other species of the genus *Volvox *(with the exclusion of the section *Volvox*) and the genera *Eudorina *and *Pleodorina *constitute another, much larger monophyletic group, the *Eudorina *group [16,17] (see Additional File 1 for details).

All volvocine algae are flagellates that are dependent on phototactic behavior because they require sufficient light for photosynthesis and therefore need to orientate themselves in an efficient manner (for review, see [18]). For this, each (somatic) cell of each volvocine alga has two flagella for locomotion and an eyespot apparatus for light perception. The photoreceptors involved in the photoresponse have been identified both in *Chlamydomonas reinhardtii *[19-22] and *Volvox carteri *[23,24]. These photoreceptors are integral parts of a primordial visual system, forming, with the pigmented eyespot, a functional eye, which seems to share the same ultrastructure and physiology in both the unicellular *Chlamydomonas *and the multicellular *Volvox *[5,25-28].

In fact, the somatic cells of *Volvox *and its relatives are called "*Chlamydomonas*-like" in countless publications particularly due to the similarity of their eyes (and due to the presence of two flagella). *Volvox*, however, is much more than a multicellular version of *Chlamydomonas*. To allow for effective forward swimming and phototaxis, multicellular organization created two major requirements: (1) the appropriate arrangement and orientation of the biflagellated cells and (2) the coordination of cell behaviors.

Initially, a spherically symmetric arrangement and orientation of identical biflagellated cells might appear to be an ideal configuration for a multicellular organism. A hypothetical 5000-celled, spheroidal *Chlamydomonas*, however, would not be able to display any effective motility, not to mention phototaxis [29]. Forward swimming would be impossible because each cell in such a spherical surface monolayer would have a counterpart on the opposite side of the spheroid with flagella pointing in the opposite direction, and therefore the flagellar forces generated by opposing cells would neutralize each other. Because *Volvox *is actually a spheroidal organism, questions arise as to how it actually swims forward and what is different from the swimming motion of *Chlamydomonas*. Although *Chlamydomonas *and *Volvox *swim forward and rotate simultaneously, they have to do it in a very different manner owing to their different geometries and complexities. *Chlamydomonas *swims toward the cellular anterior with a breaststroke-like motion and rotates counterclockwise (as viewed from behind the cell) about the cellular anterior-posterior axis [30-35]. It switches between nearly straight-ahead swimming with synchronous beating and abrupt large reorientations with asynchronous flagellar beating, which has been described as a eukaryotic version of the "run-and-tumble" motion of peritrichously flagellated bacteria [36]. Because the *Chlamydomonas *cell rolls around its longitudinal cell axis during helical forward swimming, its eye receives a sinusoidally modulated light signal. In addition to phototaxis, *Chlamydomonas *also shows photophobic responses [34,35,37,38]. The photophobic response is caused by a switch in the flagellar waveform, whereas phototaxis is caused by changing the balance of beating between the two flagella [39-41].

In the spheroidal colonial and multicellular volvocine species, the cellular anterior-posterior axis is different from the anterior-posterior axis of the whole organism. The effective strokes of both flagella beat in the same or almost the same direction toward the posterior pole of the spheroid, and they beat in parallel planes pushing the spheroid in the posterior-anterior direction [42-44]. This type of beating is indispensable for the effective forward swimming of spheroidal colonial and multicellular flagellates; it evolved together with the transition from unicellularity to multicellularity and is caused by the rotation of the basal bodies that underlie the flagella and determine their orientations [6,8,42,45]. The net effective propulsion generated by the effective strokes is not directed exactly parallel to the posterior-anterior axis of the spheroid and therefore results in rotation during forward swimming [29,44,46]; this characteristic forward rolling motion is also reflected in the name of the *Volvox *genus (Latin for "fierce roller") [47]. The arrangement, orientation, and beating direction of the biflagellated cells, however, explain only the reason that *Volvox *can swim forward but not its phototactic behavior.

For more than 100 years, scientists have studied the swimming behavior and phototaxis in multicellular (and colonial) volvocine algae (e.g., [29,46,48-61]). A key question in these studies on the phototactic behavior in multicellular volvocine algae was always how the coordination among their cells works. A cell-to-cell communication network among somatic cells that serves as the basis for the coordination of phototactic behavior seems to be rather obvious. Indeed, several species of the genus *Volvox *possess a network of cytoplasmic bridges even at the adult stage, which could allow for signal transduction between cells. However, amazingly, the multicellular volvocine algae are able to accomplish phototactic swimming without any known direct communication among cells [49,62,63]. This lack of communication becomes clear in species of the genus *Volvox *that lack any intercellular connections in adults (e.g., *V. carteri*); these species are well coordinated and swim toward the light, just as those with connections do. Likewise, individuals from species with cytoplasmic connections (e.g., *V. globator*) that had their connections microsurgically disrupted still showed phototactic behavior [49,62,63]. These observations bring up the question: How does the coordination of phototactic behavior work without direct communication among cells?

A change in the swimming direction in colonial and multicellular volvocine algae results from changes in the flagellar activity of cells in different portions of the spheroid. For *V. aureus *and *V. carteri *(both from the *Eudorina *group), fluid streamlines at the anterior pole were shown to stop transiently upon illumination, suggesting that cells at the anterior pole are responsible for the photoresponses [53-55,64]. Earlier, Fritsch [65] reported that cells at the anterior pole of the spheroid have larger eyespots than those at the posterior pole, which supported the idea of more sensitive eyes at the anterior pole [29,55]. Sakaguchi and colleagues [54,55] concluded that minor changes in the behavior of cells at the anterior pole (of *V. carteri *and *V. aureus*) that come into or out of an illumination beam path during the spheroid's swimming with rotation cause both positive and negative phototaxis.

Previously, two competing models for positive phototactic steering in *Volvox *had been proposed. In the "variable beat frequency model" [48,52-55] the cells at the anterior pole reduce their beat frequency as the light intensity changes as cells rotate from the shaded side to the one in the light. In addition, they increase their beat frequency as they rotate from the illuminated side to the one in the dark. In the "variable beat direction model" [46], the cells change their flagellar beat direction when the cells rotate from the shaded side to the one in the light and also when they rotate from the illuminated side to the one in the dark. On the dark side, the flagella beat along the anterior-posterior axis toward the posterior pole of the spheroid, whereas on the illuminated side, the flagella beat laterally. Hoops *et al. *[57] tested both models in *V. carteri*, and their results supported the variable beat frequency model while being inconsistent with the variable beat direction model for phototactic turning.

A recent mathematical theory and measurements in *V. carteri *suggest that colony rotation and photoresponsive kinetics have evolved to be mutually tuned and optimized for phototaxis [61].

In the present study, the phototactic behavior in the spheroidal, multicellular volvocine green alga *V. rousseletii *(section *Volvox*), one of the fastest swimmers among all volvocine green algae, was analyzed to address the question of how a multicellular organism accomplishes phototactic swimming without any known direct communication among cells. To our knowledge, this is the first detailed description of phototactic behavior in a species within the section *Volvox*.

In response to light stimuli, not only was the beat frequency in cells on the anterior hemisphere changed, but also the beat direction was reversed. Moreover, the flagella waveform changed when the cells reversed the beat direction in response to changes in light intensity. The pulsed illumination of somatic cells at the anterior pole demonstrated that *V. rousseletii *quickly responds to changing light conditions with a reversal of the beating direction. We show that positive phototaxis is assisted by gravitational forces and that there is no negative phototaxis in *V. rousseletii *(under our experimental conditions). Finally, we present a model for positive phototactic steering in *Volvox rousseletii *that is consistent with both our results and those of previously published studies.

## Results

### Phylogenetic analysis of *Volvox rousseletii *strain MI01

The wild-type *Volvox rousseletii *strain MI01 was isolated in 1998 from the Machi-ike pond in Oh-gata, Tsukuba-shi, Ibaraki, Japan. A phylogenetic analysis of this strain was made to confirm its identity, place it accurately within the phylogenetic tree, and confirm that it belongs to the section *Volvox*. The analysis was based on cDNA fragments of chloroplast genes (*psa*A, *psa*B and *rbc*L) and an internal transcribed spacer sequence (ITS2) from 47 volvocine species [11,16,17,66-70]. The background and procedures of the phylogenetic analysis are described in Additional File 2. Detailed information for the cDNA sequences (e.g., source species and/or strains, abbreviations, accession numbers and references) is described in Additional File 3. The alignments that were performed are shown in Additional Files 4, 5 and 6, and the sequence identity calculations are described in Additional Files 7, 8, 9 and 10. A consensus tree based on 30,000 resampled data sets for the *psa*A, *psa*B and *rbc*L cDNA fragments (10,000 data sets from each) is shown in Additional File 1, together with some phylogenetic commentary. The phylogenetic tree indicates polyphyly of the genus *Volvox*, which is consistent with several previous studies [10,12,16,17]. The species *V. barberi*, *V. globator *and *V. rousseletii *appear on the same branch, which corresponds to the monophyletic section *Volvox *[5,14,16,17]. The species used in this study, *Volvox rousseletii *strain MI01, appears next to the *Volvox rousseletii *UTEX 1862 strain.

### Polarity of the *V. rousseletii *spheroid

**Figure 1:**
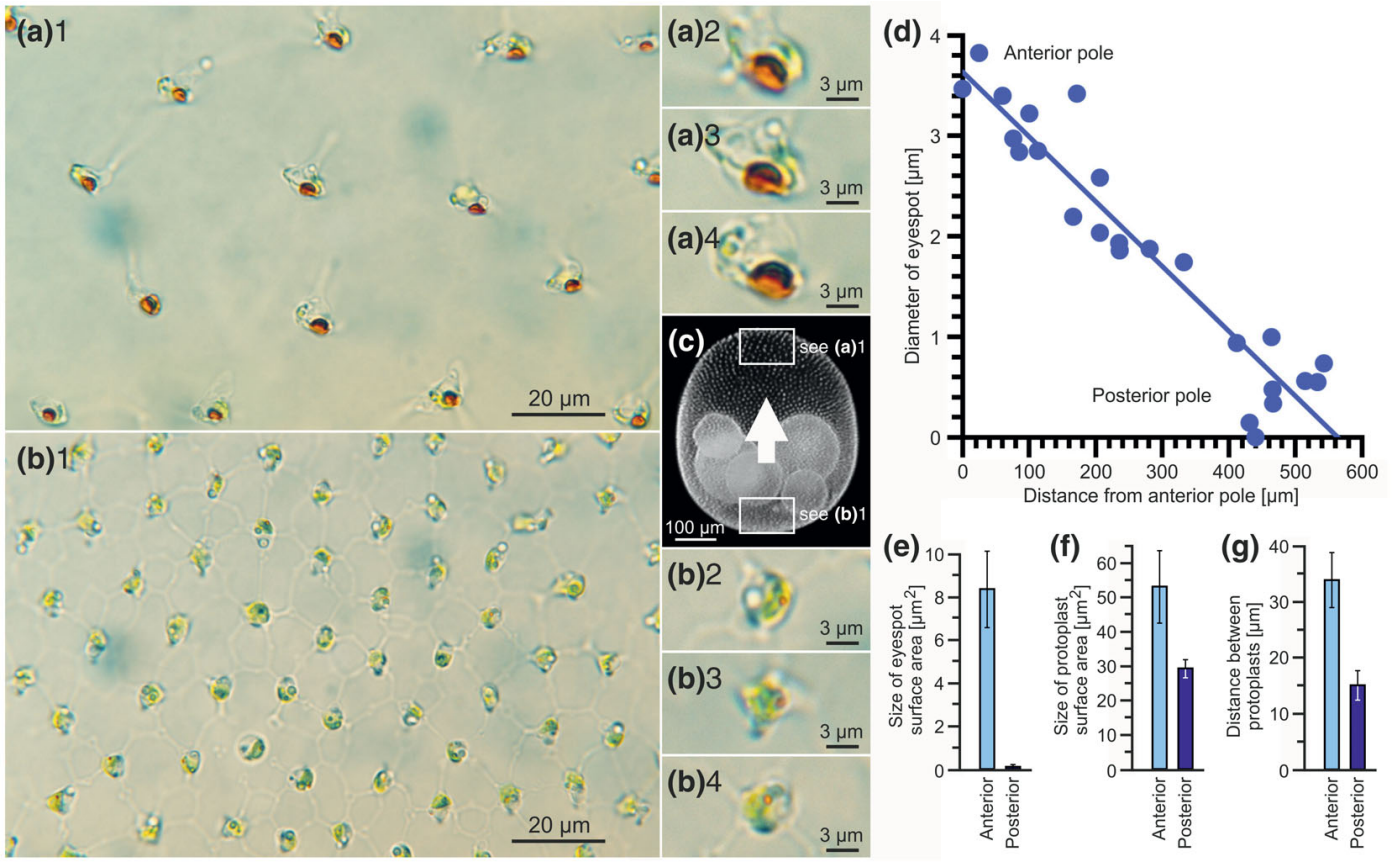
**Organismal polarity of *V. rousseletii***. **(a)**1 Closeup view of somatic cells at the anterior pole (see frame in Figure 1c). **(a)**2-**(a)**4 Individual somatic cells from the anterior pole at high magnification. **(b)**1, Closeup view of somatic cells at the posterior pole (see frame in Figure 1c). **(b)**2-**(b)**4 Individual somatic cells from the posterior pole at high magnification. **(c) **The swimming direction of *V. rousseletii *along its posterior-anterior axis is indicated by a white arrow. The progeny are within the posterior hemisphere. The polar diameter of the spheroid is always greater than the equatorial diameter. The frames indicate the areas shown in (a)1 and (b)1. **(d) **The diameter of the eyespots was plotted against the linear distances between the respective somatic cells and the anterior pole (n = 24). **(e) **Size of the eyespot surface area at the anterior and posterior poles (n = 15). **(f) **Size of the protoplast surface area at the anterior and posterior poles (n = 60). **(g) **Distance between protoplasts at the anterior and posterior poles (n = 250). **(e)**-**(g) **Error bars indicate the standard deviation of the *y*-value. **(d)**-**(g) **The sizes and distances were calculated from the photomicrographs.

An adult *V. rousseletii *alga consists of a spherical surface monolayer of ~5000 small biflagellate, terminally differentiated somatic cells and 1-20 asexual reproductive cells (the number greatly depends on environmental conditions). The cells are regularly positioned in a transparent spheroid of a glycoprotein-rich extracellular matrix, and this spheroid displays a distinctive morphological polarity along its anterior-posterior axis. The whole organism view shows that the spheroid is somewhat more enlarged along the anterior-posterior axis than along the equatorial axis (Figure 1c). In embryogenesis, mature asexual reproductive cells, which are located in the posterior hemisphere, undergo embryonic cleavage divisions, with the characteristic cleavage pattern of the embryo establishing the polarity of the organism. The fully cleaved embryo is inside out and must turn right side out in a gastrulation-like process called inversion [5]. Next, the juvenile spheroids within the adult increase in size by depositing large quantities of extracellular matrix and finally hatch from their parent. The juvenile spheroids are always located within the posterior hemisphere due to the position of the original reproductive cells (Figure 1c). The juvenile spheroids cause the posterior hemisphere to be denser than the anterior hemisphere [71,72], with a likely contribution by the closer spacing of somatic cells on the posterior hemisphere (see next paragraph). This anisotropic mass distribution ensures that the spheroids always sink with their posterior pole first.

**Figure 2:**
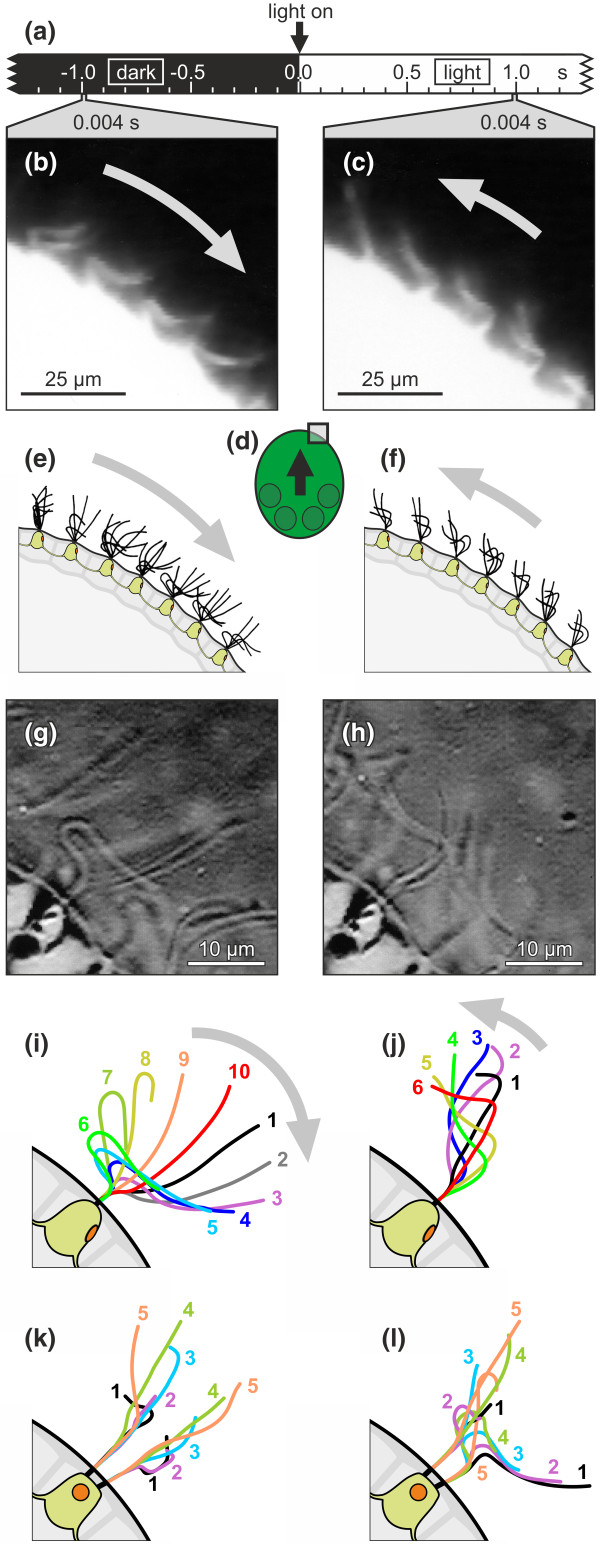
**Flagellar strokes and flagellar waveforms of the normal and reverse beating mode**. The flagellar activity of somatic cells before and after light stimulation. Light gray arrows indicate the direction of the effective flagellar strokes. **(a) **Time scale. **(b) **and **(c) **Dark-field images of somatic cells close to the anterior pole (see Figure 2d). Both images show the same cells (exposure time, 0.004 s). **(b) **The flagellar activity before the light was turned on (normal beating mode). **(c) **The flagellar activity immediately (1.0 s) after the light was turned on (light intensity: 20.8 μmol m^-2 ^s^-1^). The flagella showed the reverse beating mode. **(d) **The frame indicates the analyzed area relative to the spheroid, and the black arrow indicates the spheroid's inherent moving direction. **(e) **and **(f) **Stylized representation of the recorded flagellar strokes of somatic cells before and immediately (1.0 s) after light stimulation. **(g) **and **(h) **Frames from high-speed captured microvideographs of the same somatic cell. The viewing angle is almost perpendicular to the bending plane of the flagella. **(g) **Normal beating mode (before light stimulation). **(h) **Reverse beating mode immediately (0.1 s) after light stimulation. **(i) **and **(j) **Superimposed flagellar waveforms derived from successive stop-motion, high-speed video frames of the cell shown in (g) and (h) that show complete stroke. Only one flagellum is shown. **(i) **Normal beating mode. **(j) **Reverse beating mode. **(k) **and **(l) **Superimposed flagellar waveforms of both flagella of a somatic cell in normal beating mode. To allow for the simultaneous tracing of both flagella, the viewing angle is almost parallel to the bending plane of the flagella. The interval between the flagellar traces in (k) and (l) is only 0.13 s.

**Figure 3:**
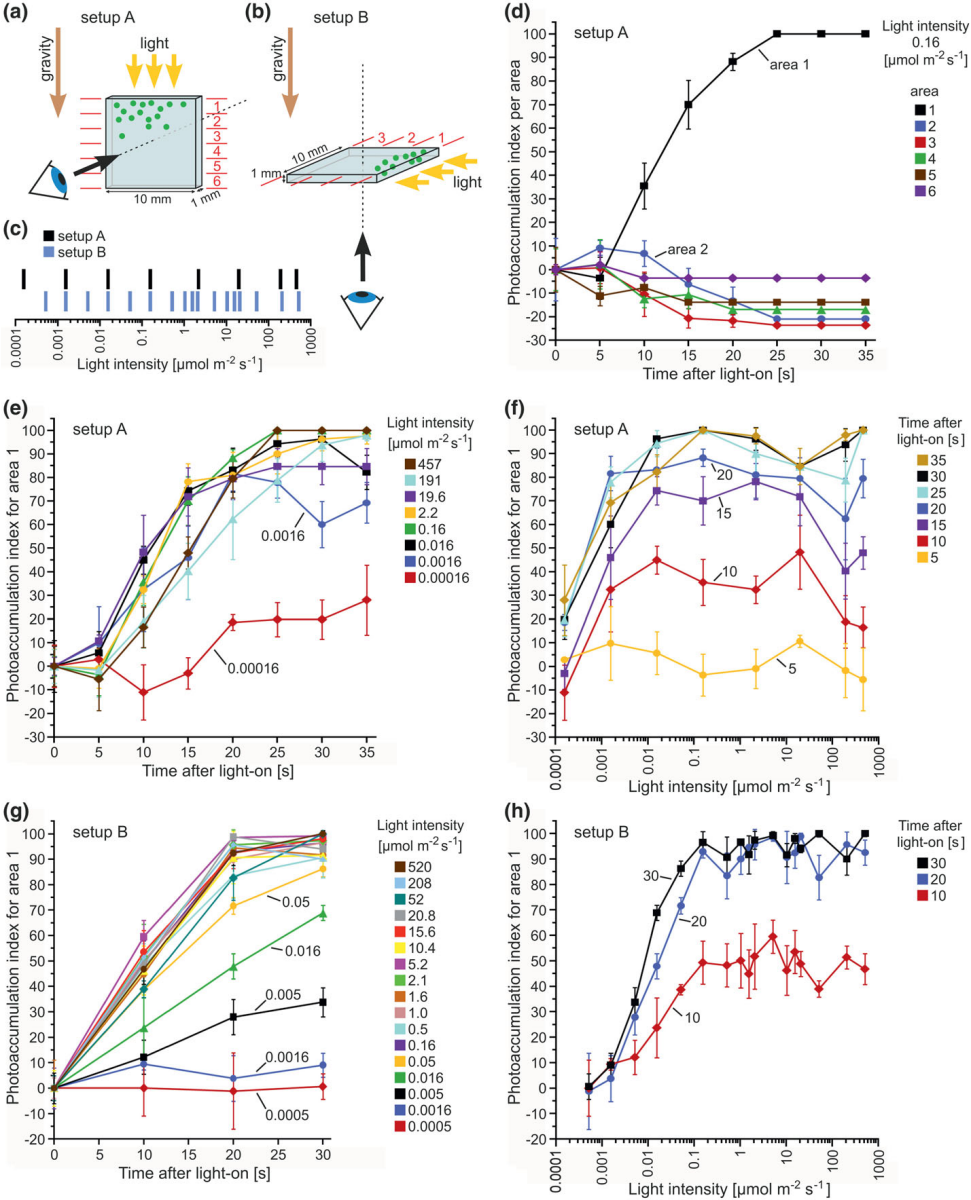
**Quantification of the phototactic movements depending on the applied light intensity**. **(a) **and **(b) **The viewing direction, the direction of the light vector and the direction of the gravity vector is indicated. **(a) **In the vertical setup A, the direction of the light vector is identical to the direction of the gravity vector. The observation chamber was divided into six areas with area 1 being closest to the light source. **(b) **In the horizontal setup B, the direction of the light vector is perpendicular to the direction of the gravity vector. The observation chamber was divided into three areas with area 1 being closest to the light source. **(c) **Schematic representation of the light intensities used in setups A and B. **(d)**-**(h) **Phototactic movements were quantified by calculating the photoaccumulation index (PI). The higher the PI value, the stronger the photoaccumulation. **(d) **The PI was calculated separately for each area (setup A) and was plotted against the time after the light was turned on (light intensity: 0.16 μmol m^-2 ^s^-1^). **(e) **The PI for area 1 in setup A was plotted against the time after the light was turned on using eight different light intensities. **(f) **The PI for area 1 in setup A was plotted against the different light intensities. Seven different times were analyzed separately at intervals of 5 s. **(g) **The PI for area 1 in setup B was plotted against the time after the light was turned on using 17 light intensities. **(h) **The PI for area 1 in setup B was plotted against the different light intensities. Three different times were analyzed separately at intervals of 10 s. **(d)**-**(h) **The color assignment is indicated. Error bars refer to the standard deviation of the *y*-value (n = 3).

**Figure 4:**
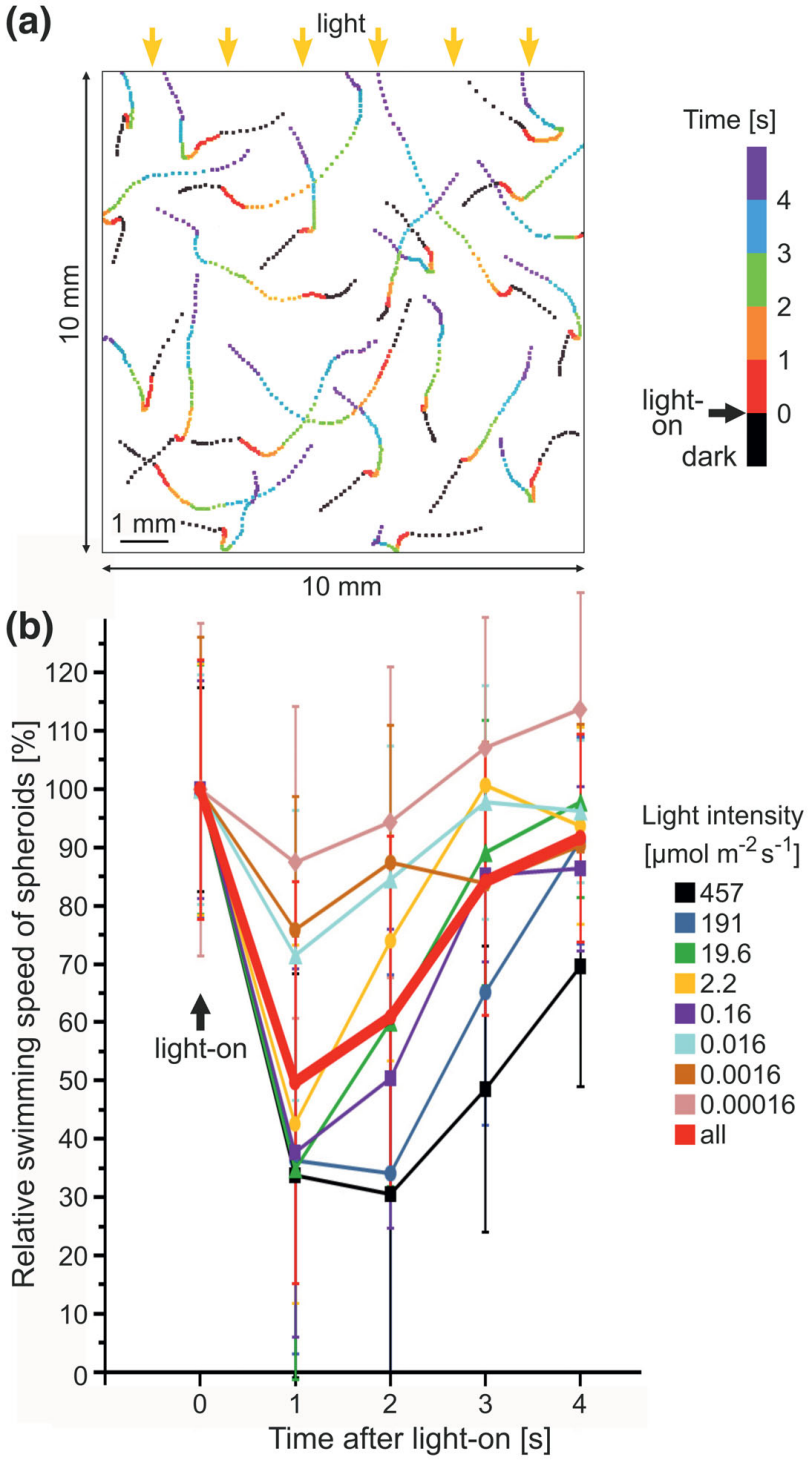
**Swimming speed of *V. rousseletii *spheroids after a dark-light switch, depending on the light intensity**. **(a) **An example of the tracks of the spheroids in the observation chamber (vertical setup A, Figure 3a). The position of each spheroid was recorded every 0.1 s starting at 1 s before the dark-light switch. The color of the position marks changed every second as indicated. The yellow arrows indicate the light source (unidirectional illumination). **(b) **The relative swimming speed of spheroids was plotted against the time after the light was turned on (vertical setup A, Figure 3a). The swimming speed immediately before light stimulation was set to 100%. A total of eight different light intensities were analyzed separately, and the color assignment is indicated. In addition, the average values for all intensities are given (bold red line). Error bars refer to the standard deviation of the *y*-value (n = 20).

Only volvocine algae in the section *Volvox*, to which *V. rousseletii *belongs, retain robust cytoplasmic bridges even in the adult [5,14]. An extensive network of these characteristic cytoplasmic bridges makes the somatic cells appear stellate when viewed from their flagellar ends (Figs. 1a, 1 to 4, and 1b, 1 to 4) and bell-shaped when viewed from the side (shown schematically in Figs. 2i to 2l). The somatic cells at the anterior pole are larger than those at the posterior pole, and the cell-to-cell distances at the anterior pole are also larger (Figure 1, a1 and 1, b1). Owing to the transparent extracellular matrix, the outer edge of each somatic cell is indistinct; therefore, we measured the protoplast surface areas and distances between protoplasts. When compared with the posterior pole, the protoplast surface areas and the distances between protoplasts at the anterior pole were 1.8-fold larger (Figure 1f) and 2.3-fold longer (Figure 1g), respectively. The decrease in cell distances along the anterior-posterior axis also becomes apparent in the view of the whole spheroid (Figure 1c), and as mentioned above, this closer spacing of somatic cells on the posterior hemisphere might contribute to the higher density of the posterior hemisphere. The somatic cells at the anterior pole have large eyespots (~3.6-μm diameter) that face toward the posterior pole (Figure 1a, 1 to 4). The diameter of the eyespots decreased continuously along the anterior-posterior axis (Figure 1d). Cells at the posterior pole have either a tiny or an undetectable eyespot. Those eyespots that are present do not face in the same direction (Figure 1b, 1 to 4). A comparison of the eyespot surface areas of cells at the anterior pole with those at the posterior pole highlights these differences in eyespot size; the surface areas at the anterior pole are ~80-fold larger than those at the posterior pole (Figure 1e), indicating that the cells at the anterior pole possess the highest light sensitivity and that the sensitivity decreases continuously along the anterior-posterior axis toward the posterior pole. The cells at the posterior pole are light-insensitive and essentially blind.

### Efficiency of phototactic movements as a function of light intensity

The phototactic movements of the *V. rousseletii *spheroids were analyzed in vertical (setup A, Figure 3a) and horizontal directions (setup B, Figure 3b) under continuous unidirectional illumination with a broad range of intensities spanning approximately eight orders of magnitude (Figure 3c). For the calculation of the photoaccumulation index (PI), the observation chamber was divided into six (setup A) or three (setup B) areas, with area 1 closest to the light source.

An example of the calculation of the PI for all areas at 0.16 μmol m^-2 ^s^-1 ^in setup A is shown in Figure 3d. When the light is on, the PI for area 1 remains relatively constant for ~5 s and then increases continuously. The initial delay is caused by a reduction in swimming speed after the dark-light switch (see below sections "Swimming speed after a dark-light switch as a function of light intensity" and "Swimming speed after light stimulation as a function of the swimming direction"). The PI for area 1 reaches its maximum after ~25 s, whereas the PI of the other areas decreases in return. The PI for area 2 increased temporarily because the spheroids have to cross this area to reach area 1. In Figures 3e to 3h, only the results for area 1 are shown because they sufficiently describe the photoaccumulation.

In the vertical setup A, light intensities of 0.016 μmol m^-2 ^s^-1 ^or above resulted in the same phototactic behavior as described above (Figure 3e and 3f). Remarkably, high light intensities of up to 457 μmol m^-2 ^s^-1 ^also yielded strong positive phototaxis, with the spheroids accumulating in area 1 within ~25 s. Even very low light intensities (0.00016 μmol m^-2 ^s^-1^) resulted in a small increase of the PI. The photoaccumulation in the horizontal setup B (Figure 3g and 3h) was essentially the same as in the vertical setup. In contrast, very low light intensities (i.e., 0.0016 μmol m^-2 ^s^-1 ^or below) had no significant effect. The lower sensitivity at low light intensities in setup B compared with setup A seemed to be caused by the facilitating effects of gravity on phototaxis (see sections "Swimming speed after light stimulation as a function of the swimming direction" and "Gravity facilitates positive phototaxis" in the Discussion), which might not be observed in setup B due to the dimensions of the observation chamber combined with the direction of the light vector. At light intensities between 0.005 and 0.16 μmol m^-2 ^s^-1^, the PIs at 10, 20, or 30 s after the light is switched on increase with increasing light intensity (Figure 3h), such that the higher the intensity, the earlier the spheroids reach the light source. There is no further increase in PI at intensities above 0.16 μmol m^-2 ^s^-1 ^(Figure 3h). As in setup A, even the high light intensities (up to 520 μmol m^-2 ^s^-1^) produce a strong positive phototaxis. These results indicate that there is no negative phototaxis in *V. rousseletii *(under the conditions of this study).

### Efficiency of phototactic movements after a longer dark period

To determine whether the length of the dark period before the light stimulus influences the phototactic movements, light-adapted spheroids were kept in the dark for 0 to 90 min and subsequently subjected to a photoaccumulation assay (Table 1). These results demonstrated that longer dark periods reduced the efficiency of the phototactic movements, decreasing continuously from 100% to ~65% within an hour in the dark. Periods of darkness longer than 1 hour, however, did not result in any further decrease of significance.

**Table 1:** Reduced efficiency of the phototactic movements after a longer dark period

Period of Darkness Before Illumination (min)	Spheroids in Area 1* (%)	SD (%)
0	100	2.5
0-10	97.3	3.1
10-20	86.5	8.7
20-30	87.7	3.3
30-40	84.5	1.3
40-50	81.5	4.1
50-60	67.2	11.7
60-70	65.2	6.1
70-80	63.8	11.1
80-90	61.9	13.6

*The duration of the photoaccumulation assay was 30 s (n = 3 for each time period). The number of light-adapted spheroids accumulated in area 1 (setup B, Figure 3b) was set to 100%. These light-adapted spheroids correspond to 0 min in the dark. The light intensity during both the light adaptation and the photoaccumulation assay was 2.08 μmol m^-2 ^s^-1^.

### Swimming speed after a dark-light switch as a function of light intensity

To determine the swimming speed of individual spheroids after a dark-light switch, the tracks of the spheroids were recorded as shown in Figure 4a. The speed of individual spheroids was determined at 1-s intervals from 1 s before to 4 s after the dark-light switch. Eight different light intensities, spanning approximately eight orders of magnitude, were analyzed separately, and the relative swimming speed of the spheroids was plotted against the time after the light was turned on (Figure 4b). A dark-light switch using low light intensities (i.e., between 0.00016 and 0.016 μmol m^-2 ^s^-1^) caused a speed reduction to ~70% relative to the swimming speed immediately before light stimulation. Higher light intensities (i.e., between 0.16 and 457 μmol m^-2 ^s^-1^) caused a speed reduction to 35% to 45% (Figure 4b). These speed reductions occurred within 1 s of the onset of illumination, and the former speed before the onset of darkness was recovered in ~3 to 4 s. This temporary speed reduction reflects a brief photophobic response of the organism. Overall, the results with all intensities indicate that the higher the light intensity, the stronger the speed reduction.

The absolute swimming speed of recently hatched spheroids in the dark (setup A, Figure 3a) was determined to be ~880 μm s^-1 ^(SD 270 μm s^-1^, n = 50) in an upward direction and 1060 μm s^-1 ^(SD 440 μm s^-1^, n = 50) in a horizontal direction. In contrast, Solari and coworkers [73,74] reported an upward swimming speed of ~500 μm s^-1 ^(up to ~590 μm s^-1^) for *V. rousseletii*. These differences may be due to different culture conditions and a different experimental setup, particularly with respect to the dimensions of the observation chamber. In our experiments, the spheroids were only able to swim along the *x*- and *y*-axes but not the *z*-axis, which extended only 0.1 cm. Solari *et al. *used a chamber that extended 1 cm along the *z*-axis, allowing movements along this axis that may account for the lower values for the upward swimming speed (*y*-axis).

### Swimming speed after light stimulation as a function of the swimming direction

**Figure 5:**
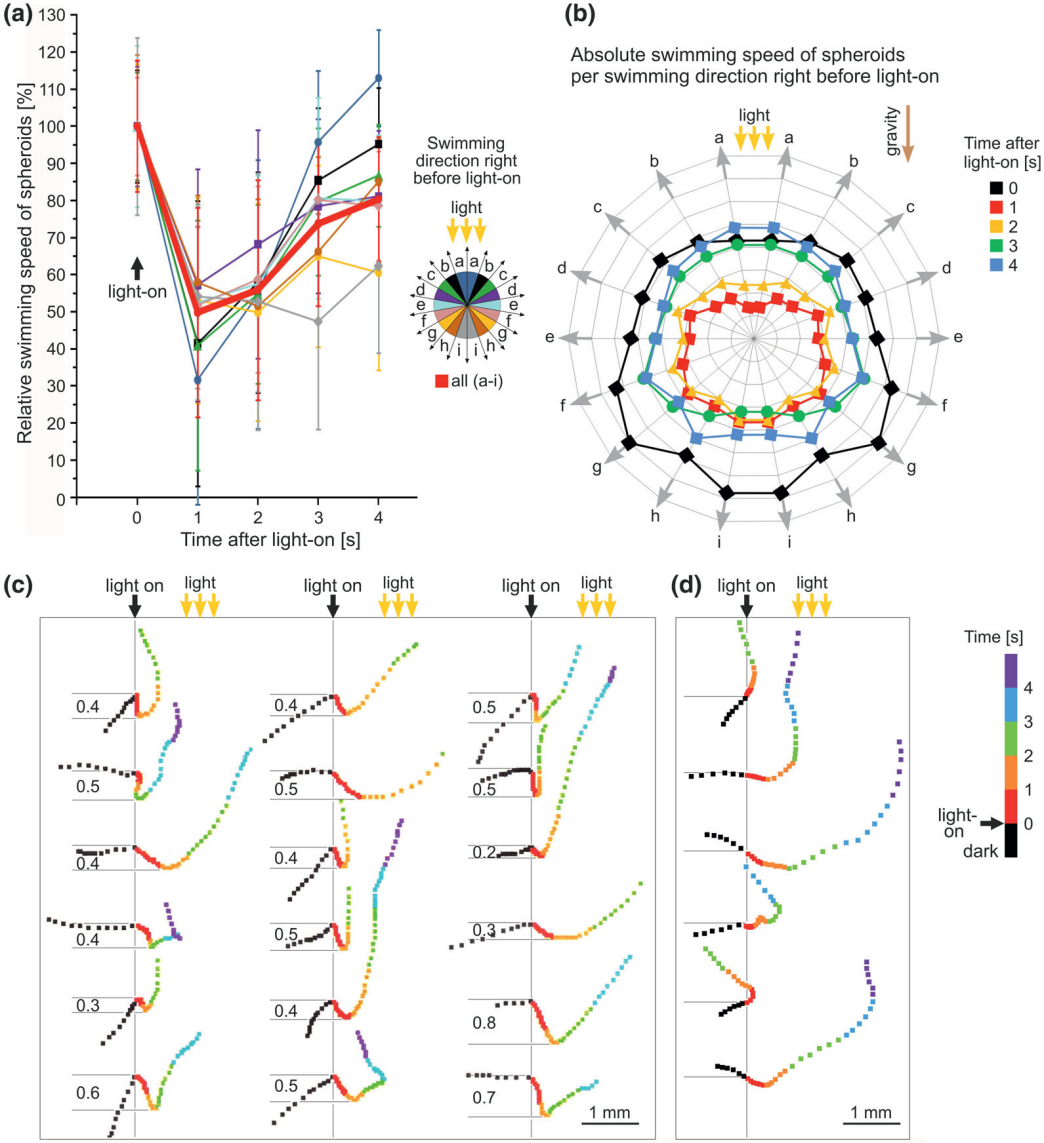
**Swimming speed after a dark-light switch, depending on the swimming direction**. **(a) **The relative swimming speed of the spheroids was plotted against the time after the light was turned on (vertical setup A). The swimming speed immediately before light stimulation was set to 100%. The swimming direction relative to the light source immediately before light stimulation was recorded and assigned to different sectors (a-i), and the color assignment is indicated. Each sector was analyzed separately. In addition, the average values for all directions are given (bold red line). Error bars refer to the standard deviation of the *y*-value (n = 20). **(b) **The swimming direction relative to the light source immediately before light stimulation was recorded and assigned to different sectors (gray arrows, a-i). The absolute swimming speed of the spheroids is shown for 0-4 s after the light was turned on (vertical setup A). All swimming speeds were plotted against the swimming direction immediately before light stimulation. The color assignment, the direction of the light vector, and the direction of the gravity vector are indicated. Reading from the inside outward, the gray concentric circles indicate 0.2, 0.4, 0.6, 0.8, 1.0, 1.2, 1.4, and 1.6 mm s^-1^. **(c) **and **(d) **The typical tracks of spheroids after a dark-light switch. The tracks were aligned relative to the moment of the dark-light switch (vertical lines), and the indicated color assignment reflects the time before and after the dark-light switch. **(c) **Tracks from vertical setup A, with the sinking depth of spheroids right after the dark-light switch indicated (in mm). **(d) **Tracks from horizontal setup B. **(a)**-**(d) **The yellow arrows indicate the light source. The light intensity was 2.1 μmol m^-2 ^s^-1 ^(intensities up to 457 μmol m^-2 ^s^-1 ^produced the same results).

To investigate the swimming speed of individual spheroids after a dark-light switch relative to the swimming direction just before the light (2.1 μmol m^-2 ^s^-1^) was turned on, setup A (Figure 3a) was used, and the spheroids were tracked as described above (Figure 4a). In each track, a line between the spheroid's position 1 s before the dark-light switch and its position right at the dark-light switch was drawn. The angle between each of these lines and the direction of the light vector was determined, and the angles were assigned to sectors a-i (see legend in Figure 5a). The speed of each spheroid was determined at 1-s intervals from 1 s before to 4 s after the onset of illumination. For each swimming direction in the dark (a-i), the relative swimming speed of the spheroids was plotted against the time after the onset of illumination (Figure 5a). The dark-light switch caused a speed reduction, regardless of the directions in which the spheroids were swimming immediately before the dark-light switch. This result is even true for direction a, in which the spheroids had already swum to the light source by chance as the light was turned on. There were, however, sector-dependent differences in speed reduction. For sectors a-c, speed was reduced to ~30% to 45% compared with the swimming speed immediately before light stimulation, and for sectors d to i, it was reduced to ~50% to 60% (Figure 5a). These sector-dependent differences become even clearer when the absolute swimming speeds are represented in a sector-wise manner (Figure 5b). There is already a sector dependency of the absolute swimming speed before the onset of illumination (0 s, Figure 5b), indicating that spheroids swim ~1.5 times faster to the bottom than to the top. This sector-dependency remained when the light was switched on, and the absolute speed was promptly reduced (1 s, Figure 5b). Interestingly, the sector dependency persists when the spheroids accelerate to their previous speed (2-4 s, Figure 5b). Consequently, the deceleration and acceleration of spheroids after a dark-light switch is independent from the swimming direction relative to the light source immediately before the onset of illumination, but it is influenced by gravitational force.

The analysis of the course of the tracks also made the impact of gravity apparent. Tracks from vertical setup A (Figure 3a) showed that the immediate speed reduction after the dark-light switch was accompanied by a change in the moving direction toward the bottom (Figure 5c). This sinking takes ~2 s, and the spheroids drop by ~0.45 mm (SD 0.15 mm, n = 20) before they accelerate and swim to the light source. This temporary sinking is indicative of the brief photophobic response of the organism after the dark-light switch. Tracks from horizontal setup B (Figure 3b) also show an immediate speed reduction after the dark-light switch with subsequent acceleration as the spheroids swim to the light source (Figure 5d). Owing to the dimensions of the observation chamber (0.1 cm in the vertical direction) and the viewing direction (antiparallel to the gravitational force), the sinking characteristic is not seen in the horizontal setup B (Figure 5d).

### Fluid streamlines before and after a dark-light switch

**Figure 6:**
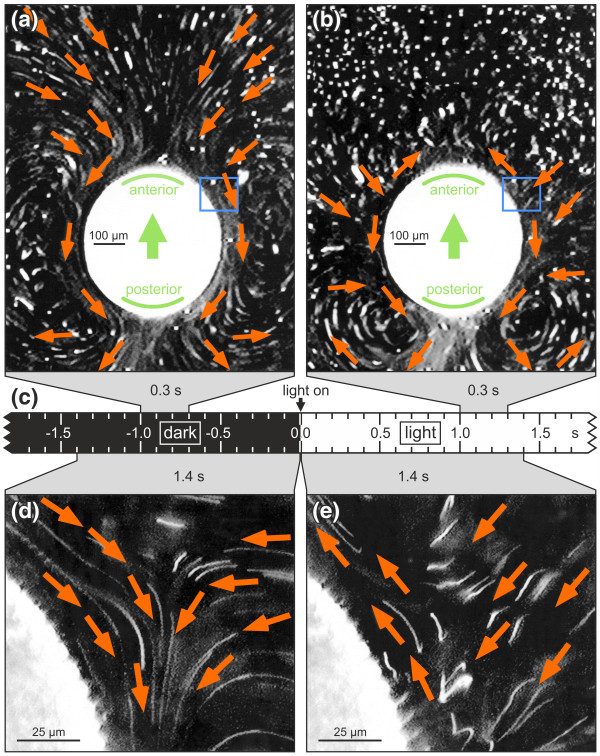
**Fluid streamlines around a *V. rousseletii *spheroid before and after light stimulation**. The fluid streamlines using polystyrene beads were visualized and taken with long exposure times. **(a) **An exposure time of 0.3 s at 1 to 0.7 s before the light was turned on. **(b) **An exposure time of 0.3 s at 1 to 1.3 s after the light was turned on. **(c) **Time scale. **(d) **Closeup of a region near the anterior pole (see blue frame in a). An exposure time of 1.4 s at 1.4 to 0 s before the light was turned on. **(e) **Closeup of a region near the anterior pole (see blue frame in b). An exposure time of 1.4 s at 0 to 1.4 s after the light was turned on. Red arrows highlight the main streamlines, and green arrows in **(a) **and **(b) **indicate the inherent moving direction of the spheroid along the posterior-anterior axis. Light stimulation was performed with green light (2.1 μmol m^-2 ^s^-1^). The infrared light used for dark-field micrography was inactive for phototaxis. **(a)**, **(b)**, **(d) **and **(e) **show the same spheroid.

Visualization of fluid streamlines in a suspension of polystyrene beads [53] allowed the analysis of flagellar activity of *V. rousseletii *spheroids before and after a dark-light switch (Figure 6). In the dark, organisms that were gently trapped between a cover glass and a glass slide produced fluid streamlines in the anterior-posterior direction along the surface of the spheroid (Figures 6a and 6d). These streams resulted in forward swimming along the posterior-anterior axis in free-swimming spheroids. Exposure of the spheroids to illumination (2.1 μmol m^-2 ^s^-1^) caused a transient stop of the streams in front of the spheroid and a reversal of the fluid streamlines along the anterior hemisphere. In contrast, the fluid streamlines along the posterior hemisphere showed no significant change (Figure 6b). The particle velocities along the anterior hemisphere were lower in the reversed direction (Figure 6e) than in the anterior-posterior direction in the dark (Figure 6d). The reversal of the fluid streamlines indicates a reversed beating direction of flagella because the streamline pattern cannot be explained by a simple stop of flagellar beating as has been observed in *V. carteri *[54,55]. The reversal of the fluid streamlines lasts for 2 to 3 seconds immediately after the dark-light switch. The initial anterior-posterior fluid streamlines are then reestablished.

These results are consistent with the speed reduction and sinking of the spheroids after a dark-light switch described above and support the existence of a photoresponsive gradient from the anterior to the posterior pole of the spheroid, with the highest responses at the anterior and no response at the posterior.

### Flagellar strokes and flagellar waveforms before and after light stimulation

Direct observation of flagellar activity by dark-field microscopy allowed an analysis of the cellular basis of the reversal of the fluid streamlines (Figures 2a-f). The flagellar activity was recorded 1 s before and 1 s after light stimulation with an exposure time of 0.004 s (Figure 2a). In the dark, the flagella of all somatic cells beat with a ciliary-type, asymmetrical motion, which we refer to as the normal beating mode (Figures 2b and 2e). This mode consists of an effective stroke in the anterior-posterior direction and a recovery stroke in the opposite direction. After the light stimulus, the cells within the anterior hemisphere completely reverse the flagellar beating direction, which we refer to as the reverse beating mode (Figures 2c and 2f). Though the effective stroke is then in the posterior to anterior direction, the cells still beat with a ciliary-type motion. The reverse beating mode of cells on the anterior hemisphere lasts for 2 to 3 seconds, after which the cells switch back to the normal beating mode with the flagellar strokes and waveforms before light stimulation are completely identical to those 2 to 3 seconds after light stimulation. This result is consistent with the observed fluid streamlines after a dark-light switch as described above. The temporary reversal of the beating direction reflects a brief photophobic response at the cellular level.

High-speed microvideography was used to examine the flagellar strokes and waveforms of individual somatic cells more closely. Frames from the captured high-speed videos of a somatic cell on the anterior hemisphere show the normal beating mode (in the dark; Figure 2g) and the reverse beating mode (0.1 s after light stimulation; Figure 2h). The superimposed flagellar waveforms show the complete stroke cycles of one flagellum in the normal (Figure 2i) and the reverse (Figure 2j) beating modes. The reverse beating mode is only transient, with the somatic cells returning to their normal beating mode after adaptation to the light, which occurs after approximately 2 to 3 seconds. Cells in the normal beating mode show a typical base-to-tip, ciliary-type motion. The stroke area has a wide spread, and the tip of the flagellum describes a relatively wide arc during its effective stroke (Figure 2i). One stroke takes ~45 ms (frequency 22 Hz [SD 6 Hz, n = 15]) in the normal beating mode. In the reverse beating mode, one stroke takes ~25 ms (frequency 40 Hz [SD 9 Hz, n = 15]), and the cells also show a ciliary-type motion. There is, however, less bending close to the base of the flagellum and a much smaller stroke area, such that the tip of the flagellum describes only a small arc during its effective stroke (Figure 2j).

The description of flagellar waveforms requires that the angle of view be essentially perpendicular to the bending plane of the flagella. From this angle of view, only the beat of one flagellum, the one closest to the observer's eye, can be described properly. To observe both flagella of a cell at the same time, the angle of view was changed so that it was almost parallel to the bending plane of the flagella (Figures 2k and 2l). The beating direction of the two flagella of a somatic cell is roughly parallel, and the effective strokes are generally directed toward the posterior pole. There is no evidence for any spatial or temporal coordination between the two flagellar beat cycles of a somatic cell, which is indicative of the asymmetrical, ciliary-type beating or between the beat cycles of the neighboring somatic cells. An example of the strokes of a single somatic cell in the normal beating mode is shown in Figures 2k and 2l. The strokes of the two flagella shown in Figure 2l were recorded only 0.13 s after the strokes in Figure 2k without any changes in light or other conditions. A comparison of these strokes not only illustrates the asynchronous and asymmetrical beating of the flagella but also documents the three-dimensional component of the strokes. Beat cycles that were somewhat out of the normal bending plane were repeatedly observed, which may be a matter of slightly variable fluid resistance due to microturbulences caused by flagella from other cells.

### Simulation of phototactic swimming with a periodically changing light intensity due to the rotation of the spheroid

**Figure 7:**
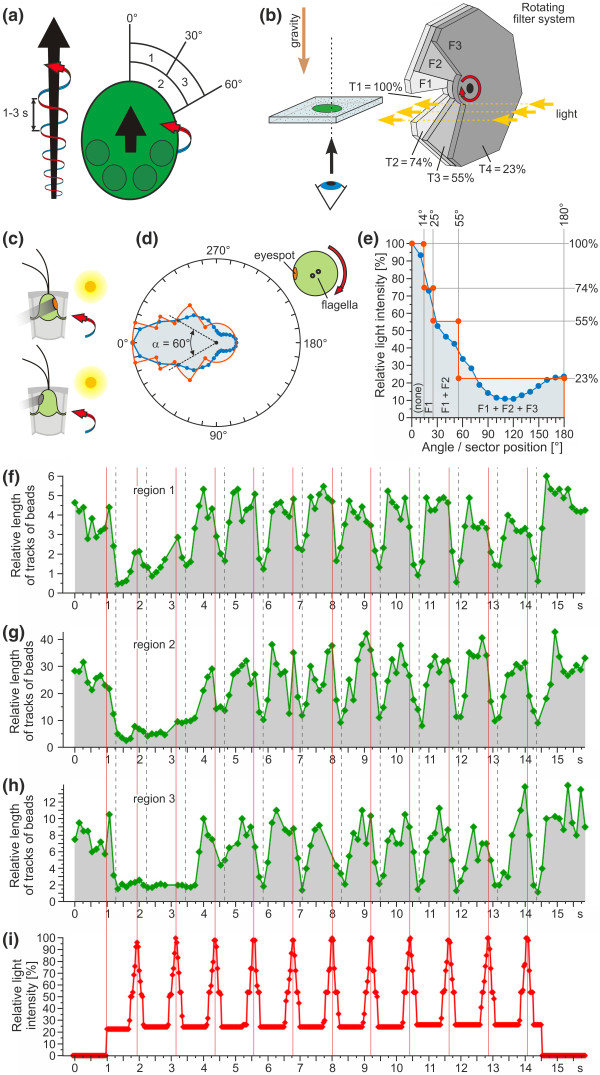
**Simulation of phototactic swimming using a rotating filter system**. **(a) **Illustration of the counterclockwise rotation as viewed from behind the spheroid of a swimming spheroid. The black arrows indicate the swimming direction. The fluid streamlines were analyzed from regions 1-3. **(b) **Experimental setup of the simulation. Filter 1 (F1): 74% transmissivity (T), 2 × 14° = 28° sector cutout; Filter 2 (F2): 74% (T), 2 × 25° = 50° sector cutout; Filter 3 (F3): 41% T, 2 × 55° = 110° sector cutout. The sandwich construction results in T1 = 100% (gap, no filter), with T2 = 74% (F1), T3 = 55% (F1 + F2), and T4 = 23% (F1 + F2 + F3). **(c) **Schematic representation of the changing light conditions in the eye during rotation. **(d) **Directivity of the eye viewed from the flagellar end of the cell. The blue line is a polar plot of tangential electric energy density at the plasma membrane overlaying the eyespot [75]. α is the half-beam width, which is the angle between the half-maximum directivities of the eye. The red line reflects the result from (e). **(e) **The blue line represents the linear plot of tangential electric energy density. Owing to the reflection symmetry, only 0° to 180° is shown. Filter combinations and the cutting of filters were calculated to match the blue line, with the most appropriate combination shown as a red line. **(f)**-**(h) **Relative length of the tracks of polystyrene beads analyzed in regions 1 to 3 using the setup shown in (b). Vertical, dotted gray lines indicate the approximate minimal length of the tracks ~300 ms after the maximum light intensity. **(i) **The light intensity was recorded by an oscilloscope. The red vertical lines in (f)-(i) indicate the maximum light intensity.

A *Volvox rousseletii *spheroid always swims along its posterior-anterior axis while rotating about its posterior-anterior axis in a counterclockwise direction (as viewed from behind the spheroid). Spheroids make one complete counterclockwise rotation approximately every 1 to 3 seconds (Figure 7a), with smaller (younger) spheroids rotating faster than larger (older) spheroids. The eyespots of somatic cells are highly directional optical devices that guide the organism to places where the light conditions are optimal for photosynthetic growth. The light signals change periodically during phototactic swimming due to the rotation of the spheroid and the directivity of the eyespot with its layers of carotenoid-filled granules below the photoreceptors in the plasma membrane [28] (Figure 7c). The periodically changing light signals of a rotating spheroid during phototaxis were simulated using a rotating filter system (Figure 7b), and fluid streamlines from three regions at the anterior pole were analyzed (Figure 7a). The construction of the rotating filter system was based on previous results on the directivity of photoreception in the unicellular alga *Chlamydomonas *[75] (Figures 7d and 7e), which corresponds to the situation in somatic cells on the anterior hemisphere of a *Volvox *spheroid (Figure 7d, blue line) as viewed from the flagellar end of the cells (Figure 7d, inset). The filter combinations and cutting of the filters were calculated to match the plot of the tangential electric energy density [75] at the plasma membrane overlaying the carotenoid-filled granules of the eyespot (Figure 7e), and the rotating filter system was constructed using the parameters given in Figure 7b. Our simulation of a spheroid swimming with rotation matches the *in vivo *situation in somatic cells very well (Figure 7d, red line). Spheroids were gently trapped between a coverglass and a glass slide, and fluid streamlines were visualized using polystyrene beads. After a dark-light switch, the light intensity was changed periodically (1.2-s intervals) with peaks in the intensity. The relative length of the tracks of polystyrene beads decreased sharply within ~300 ms after the dark-light switch (Figures 7f-h), and this period of minimal bead movement persisted for ~2.5 s, even though two peaks in light intensity occurred during this time (Figure 7i). These peaks only caused a few changes in the length of the tracks in regions 1 and 2 (Figures 7f and 7g). After this delayed reaction, which is consistent with the speed reduction and sinking of spheroids after a dark-light switch described above, the relative length of the bead tracks in all analyzed regions at the anterior pole changed with the same frequency as the fluctuations in the light intensity (Figures 7f-i). The minimal length of the tracks lagged behind the peaks in light intensity by ~300 ms. Interestingly, when the light was turned off after the periodical light pulses, the relative length of the bead tracks persisted at the maximal level (Figures 7f-i). Although the bead tracks were generally longer in region 2 (Figure 7a and 7g), which is closer to the spheroid than regions 1 and 3, the results from all three regions were quite similar.

### Reaction time after light stimulation

The reaction time of somatic cells at the anterior pole was calculated from high-speed microvideographs after a dark-light switch (Figures 2g and 2h) and from dark-field microscopy with pulsed illumination (Figure 7). High-speed microvideography demonstrated that the flagella begin to reverse the beating direction after a dark-light switch after ~100 ms (SD 25 ms, n = 5). The transition from the normal beating mode to the reverse beating mode takes ~30 ms (SD 10 ms, n = 5). Thus, the period of time spanning the start of the light stimulation to the completion of the reversed beating direction is ~100 to 160 ms. In the above experiments with periodically changing light intensities, the minimum of the fluid streamlines lagged behind the peaks in light intensity by ~300 ms (Figures 7f-i). Owing to the reaction time of ~100 to 160 ms, the reversed flagella need ~140 to 200 ms for their fluid streamlines to reach their minimum.

## Discussion

In this study, we investigated key parameters of phototactic behavior in the spheroidal, multicellular volvocine green alga *Volvox rousseletii*, one of the fastest swimmers among all volvocine green algae. This is the first detailed description of phototactic behavior in a species within the Volvocales section *Volvox*.

### Light-induced reversal of the ciliary-type beating in *Volvox rousseletii*

The reversal of the ciliary-type beating of the flagella shown in this study (Figure 2) has not yet been observed in other plants or green algae, but it has been reported for ciliates [76,77] and some metazoa [78,79]. In particular, the ciliate *Paramecium caudatum *has been widely known for its avoidance reaction, which is a sudden change in the swimming direction of the cell mediated by a transient reversal of its ciliary activity [80]. In *Paramecium*, the orientation of ciliary beating is controlled by the concentration of intracellular calcium [81-84]. Calcium is also well known for its role in the light-dependent responses of the unicellular *Chlamydomonas*, the best-studied volvocine alga with respect to flagellar activity and photoresponses [30,39,40,58,85-97]. The molecular mechanisms of light perception signal transduction and the control of flagellar beating identified in *Chlamydomonas *most likely are not specific to this species and instead are quite similar in all volvocine algae, given their close phylogenetic relationships. There are significant differences, however, in the mode of flagellar beating within the volvocine algae. Although the normal beating mode is a ciliary-type, asymmetrical motion, the light-induced flagellar beating pattern varies. *Chlamydomonas *responds with a flagellar-type, symmetrical beating, *Volvox aureus *[54] and *V. carteri *[55] respond with a cessation of flagellar beating, and *V. rousseletii *responds with a ciliary-type, asymmetrical beating in a reversed direction (Figure 2). These striking differences within the genus *Volvox *probably substantiate the polyphyletic origin of this genus. Phylogenetically, *V. aureus *and *V. carteri *belong to clade A within the *Eudorina *group [16,17], whereas *V. rousseletii *belongs to section *Volvox *[10,12,14-16] (see Additional File 1). Therefore, the light-induced ciliary-type, asymmetrical beating in a reversed direction might be characteristic of those species belonging to the section *Volvox*. The reversal of the beating direction in light-induced cells should allow for faster changes in the swimming direction of the organism and better maneuverability compared with a simple cessation of flagellar beating in light-induced cells. Thus, *V. rousseletii *should outperform *V. aureus *and *V. carteri *in phototaxis merely because of its enhanced steering ability, not to mention its faster swimming speed. This assumption still requires experimental verification.

### Gravity facilitates positive phototaxis

In addition to the flagellar forces, gravitational forces participate in phototaxis. Whenever a *V. rousseletii *spheroid slows down or stops swimming, gravity causes the spheroid to sink toward the bottom with its posterior pole first (see sections "Polarity of the *V. rousseletii *spheroid" and "Swimming speed after light stimulation as a function of the swimming direction" and Figure 5c). Even during normal swimming, gravity pulls on the posterior hemisphere more as a result of an anisotropic mass distribution caused by the denser daughter spheroids and probably by the closer spacing of somatic cells within the posterior hemisphere. This gravity effect has been quantitatively measured through biophysics in other species of the genus *Volvox*, which was referred to as "bottom-heaviness" [71,72]. Thus, through gravity, the posterior-anterior axis tends to become oriented in the opposite direction of the gravity vector such that the anterior pole tends to be on top. In the natural habitat, the sun, which serves as the light source, essentially shines from above, resulting in the direction of the light vector being roughly the same as that of the gravity vector. Therefore, gravity facilitates positive phototaxis because it brings the spheroids into the optimal orientation to swim toward the light source.

Even in the unicellular relative *Chlamydomonas*, cells with immobilized flagella sediment with their anterior ends pointing up [98,99] because the posterior chloroplast-containing part of the cell body is denser than the anterior flagella-bearing part [100].

Thus, the utilization of gravity to ensure that the anterior end is always oriented toward the top while sinking seems to be a clear evolutionary advantage for all of the phototrophic and positively phototactic volvocine algae.

### Previous models on organismal steering in the genus *Volvox*

In several earlier publications, observations in connection with phototaxis were combined to get an overall picture of how phototaxis works in *Volvox*. These descriptions and models of the swimming behavior in *Volvox *refer to the cells at the anterior hemisphere of the spheroid as being a crucial factor in phototactic steering and in its rotation during forward swimming to bring certain cells into or out of the light [46,50,52-55,101]. There had been different explanations as to how the individual cells on the anterior hemisphere change their flagellar activity in response to different light stimuli. Thus, the two above-mentioned competing models emerged, which were the variable beat frequency model [48,52-55,57] and the variable beat direction model [46]. A more recent report analyzed both models and suggested the variable beat frequency model was the best description of phototactic turning in *Volvox carteri *[57]. This model is supported by the decrease of both the rate of progression and the rotation of *V. carteri *spheroids in the presence of illumination [57]. In addition, the fluid streamlines near the anterior pole of *V. carteri *[55] and *V. aureus *[52-54] spheroids stopped upon illumination. The researchers, however, did not monitor flagellar beating directly but drew conclusions from the behavior of those spheroids exposed to light stimuli. In 2006, Nozaki *et al. *[17] assigned the phylogenetic position of the species *V. carteri *and *V. aureus *to clade A in the *Eudorina *group (see Additional File 1).

The variable beat direction model [46] is supported by the following quite contrary, indirect observation: Mast [46] determined that the rate of progression of an unidentified species of the genus *Volvox *decreased after illumination while its rate of rotation increased. Mast's drawings and descriptions, however, indicated that the observed *Volvox *species belonged to the section *Volvox *[17]. Hoops *et al. *[57] tried but unfortunately could not replicate the step-up and step-down illumination experiments with *Volvox rousseletii *and *Volvox capensis*, both of which belong to the section *Volvox*. Therefore, Hoops *et al. *[57] concluded that a (re-)analysis of phototactic steering in the spheroids of the section *Volvox *is important.

The phylogenetic differences between the investigated species may indicate that there are differences in phototactic mechanisms that originally led to the two different models. Because the above models are based on experiments that do not include direct observations of flagella due to the small size and rapid movement of the flagella, the significance of these models may be limited.

### Mechanisms for phototactic steering in *Volvox rousseletii*: A model

On the basis of the above and our own observations, we developed a mechanistic model that predicts the phototactic behavior in *V. rousseletii*. At the organismal level, we have shown that *V. rousseletii *spheroids respond to a sudden change in light intensity with a momentary photophobic response, reducing their speed rapidly for ~2 s and causing a gravitational sinking of the spheroids (Figures 4b and 5). They then accelerate quickly and obtain their original swimming speed while turning phototactically toward the light source (Figure 5). In this response, not all flagellated somatic cells of the spheroid participate in the same way. There is a photoresponsive gradient from anterior to posterior, with the highest light sensitivity at the anterior pole of the spheroid and no response to light at the posterior pole (Figure 6). This gradient is reflected by an eyespot gradient in which the cells with the largest and most light-sensitive eyespots are located at the anterior pole and the cells with the smallest, even nonexistent, and blind eyespots are located at the posterior pole (Figure 1). After a change in light intensity, only cells in the anterior hemisphere with an adequately sized eyespot perceive the change in light intensity and switch transiently from the normal beating mode to a reverse beating mode (Figure 2), which is reflected by a momentary photophobic response at the cell level. The switch in the beating direction generates a force in the opposite direction on the anterior hemisphere, while cells on the posterior hemisphere still beat in the normal beating mode, causing a deceleration of the spheroid. The subsequent acceleration and phototactic turn toward the light source is complicated by the rotation of the spheroids in a counterclockwise direction as they swim along their posterior-anterior axis (Figure 7a). In addition, the eyes of the somatic cells show a strong directivity (Figure 7d). Because the flagellar reaction cannot be observed in free swimming and rotating spheroids, the situation was simulated by holding the spheroid in place while the illumination was changed periodically (Figures 7f-i). Thus, the individual somatic cells of the anterior hemisphere are in the same situation as the corresponding somatic cells of a free swimming, rotating spheroid, which is illuminated on the side. The somatic cells of the anterior hemisphere respond quickly and periodically by reversing the direction of the flagellar beat (Figs. 2 and 7). In a free swimming, rotating spheroid, the local reversal of the beating direction causes an unbalanced force between the illuminated and the shaded side of the spheroid. As a result, the spheroid turns toward the light source. This mechanism does not require any communication between the cells because the flagellar responses of the somatic cells are coordinated only via the environmental factors of the position of the light source combined with the effects of gravity.

One final point that should be mentioned is that while each individual somatic cell has a forward and a reverse gear (Figure 2), the whole organism only has a forward gear, such that it can only swim in a posterior to anterior direction (Figure 6) but can turn easily.

**Figure 8:**
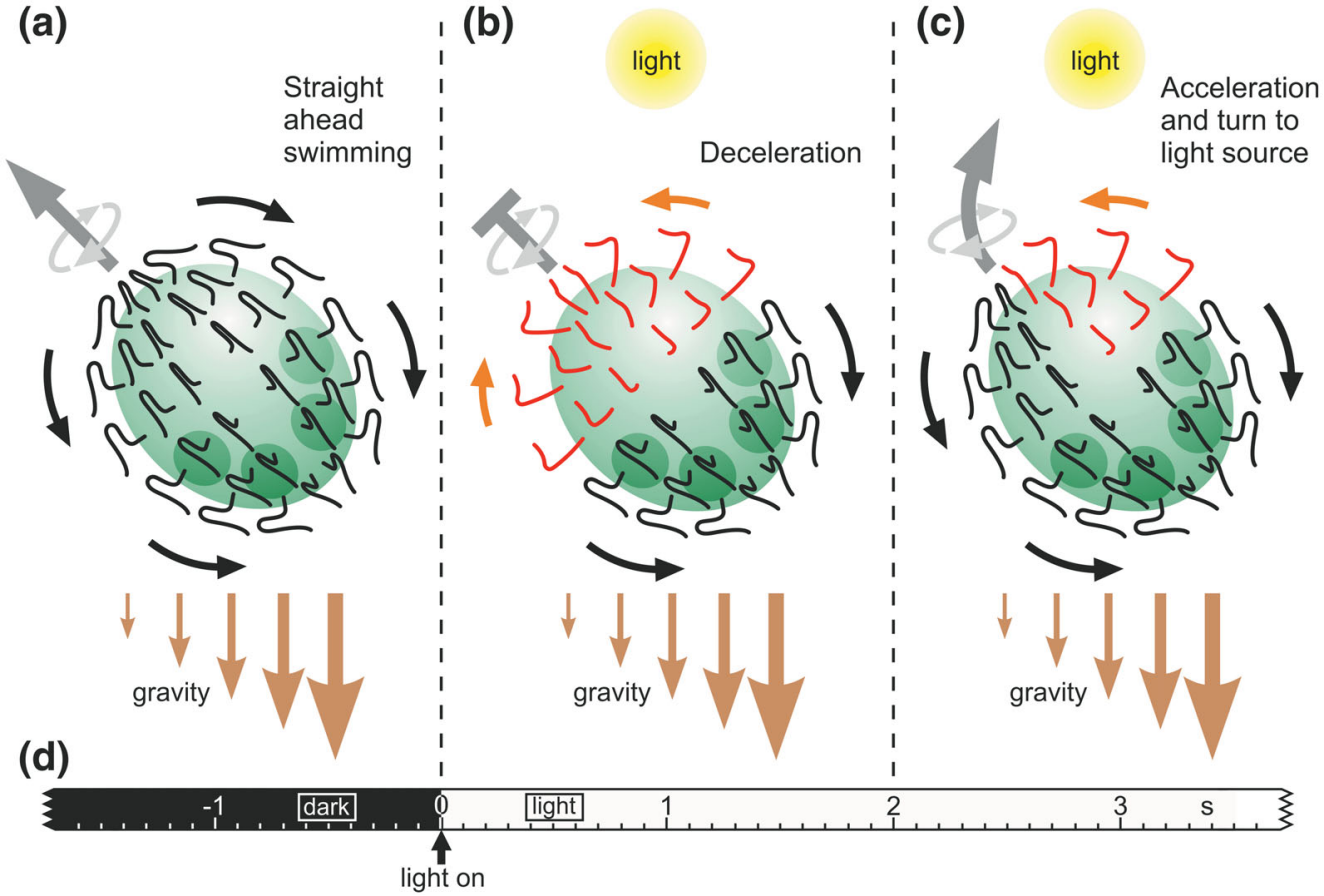
**Schematic representation of the phototactic movements in *V. rousseletii***. **(a) **Straight-ahead swimming in the dark. **(b) **A sudden dark-light switch causes the flagellar beating to reverse in the anterior hemisphere and the deceleration of the spheroid's forward movement (photophobic response). **(c) **After approximately 2 seconds, only cells on the illuminated side of the anterior hemisphere of the rotating spheroid show the reversed flagellar beating direction, resulting in an acceleration of the spheroid's forward movement and turning toward the light source. Gravity assists the phototactic movements because it pulls more on the posterior hemisphere due to an anisotropic mass distribution caused by the denser daughter spheroids within the posterior hemisphere and probably also by the closer spacing of the somatic cells in the posterior hemisphere. **(d) **Time scale.

The main components of our model of phototactic movements in *V. rousseletii *are illustrated in Figure 8, and the following conclusions summarize the relevant aspects.

## Conclusions

On the basis of all results presented herein, we developed a mechanistic model that predicts phototactic behavior in *Volvox rousseletii *and shows how this multicellular organism achieves coordination among its individual biflagellated cells without direct communication between these cells.

The model encompasses the 10 following points:

1. The *V. rousseletii *spheroids (under the conditions of this study) show only positive phototaxis but not negative phototaxis, even at high light intensities (Figure 3).

2. Spheroids swim along the posterior-anterior axis and simultaneously rotate about this axis in a counterclockwise direction, with one turn every 1 to 3 seconds (Figure 7a).

3. The spheroid has a distinctive morphological polarity along the anterior-posterior axis, with only the somatic cells at the anterior pole having large eyespots (~3.6-μm diameter) and a continuous decrease in the eyespot size toward the posterior pole (Figure 1).

4. The spheroids show a strong photoresponse gradient from the anterior to the posterior pole, and only cells in the anterior hemisphere show an effective light response (Figure 6).

5. In response to light stimuli, the somatic cells of the anterior hemisphere can reverse and recover their flagellar beating mode quickly, within ~100-160 ms (Figure 2).

6. The flagella show a ciliary-type, asymmetrical beating in both directions, but the flagella waveform and beat frequencies are different (Figure 2).

7. Gravity facilitates positive phototaxis due to the anisotropic mass distribution with the posterior of the spheroid being heavier than the anterior that results in the anterior, which contains the larger eyespots, always facing the top, where the light source normally is located (Figures 8a-c).

8. A sudden increase in light intensity causes the somatic cells of the anterior hemisphere to show a photophobic reaction for ~2 s, with an accompanying transient reversal of the ciliary activity (Figures 2c and 2h; Figure 6b), causing a deceleration and sinking of the spheroid (Figures 4, 5, and 8b).

9. During phototactic swimming, only somatic cells of the anterior hemisphere with eyespots pointing toward the light source reverse the beating direction in the presence of illumination, resulting in a turn toward the light source (Figures 2, 6, 7, and 8c).

10. The coordination of the spheroid's individual biflagellated cells during phototactic swimming occurs via environmental factors (e.g., the position of the light source and the effects of gravity) without any direct communication between the cells.

On the basis of our model of phototactic movements of the multicellular alga *V. rousseletii*, and taking into account previous considerations by Hoops [29] and Kirk [6], we conclude that the following evolutionary innovations were required to allow for phototactic movements after the transition from a unicellular *Chlamydomonas*-like ancestor to the multicellular *V. rousseletii*:

1. Both flagella of all cells must beat in essentially the same direction rather than the previously used breaststroke.

2. An extracellular matrix must hold all cells of the multicellular organism in the appropriate places on the surface of a spheroid.

3. The multicellular organism must not consist of identical cells and requires a distinctive morphological polarity, the flagella of all cells must beat toward the posterior, and the anterior cells must show a stronger light sensitivity, mediated through larger eyespots, than posterior cells.

4. The flagellar beating must cause rotation of the spheroid instead of rotation of the cell.

5. Light stimuli must cause a transient ciliary-type, asymmetrical beating in the reverse direction instead of a flagellar-type, symmetrical beating.

More work is clearly necessary, however, to confirm these evolutionary considerations.

## Methods

### Strain and culture conditions

The wild type *Volvox rousseletii *strain MI01 was grown in VT [102,103] or VTAC [104,105] medium at 20°C in a 10 h dark/14 h cycle under cool fluorescent white light at an average of 30-40 μmol photons m^-2 ^s^-1 ^of photosynthetically active radiation.

### Analysis of photomovements and tracking of *Volvox *spheroids

An observation chamber with internal dimensions of 10 × 10 × 1 mm was constructed as follows: (1) an acrylic cuvette (Sarstedt, Nümbrecht, Germany) was cut into slices with a 1-mm thickness, (2) a slice of the cuvette was glued onto a standard glass slide, and (3) the chamber was filled with a suspension of algae and covered with a coverglass, which was held in place by the adhesion force of water. The observation chamber contained 20-60 spheroids when it was used in the vertical orientation (Figure 3a) and 50-80 spheroids when in the horizontal orientation (Figure 3b). For observation in the vertical orientation (Figure 3a), the chamber was mounted upright on a stand and illuminated with a slide projector (Master Auto Lux-H; Rikagaku Seiki, Tokyo, Japan), and the movement of spheroids was recorded with a charge-coupled device (CCD) camera (XC-77; Sony, Tokyo, Japan) equipped with a C-mount macroscopic lens (Cine-Nikkor 1: 1.8, f = 25 mm; Nikon, Tokyo, Japan). For observation in the horizontal orientation (Figure 3b), the observation chamber was monitored using the CCD camera (XC-77; Sony) on a standard inverted microscope (TMS-F; Nikon). A phototactically neutral field illumination was achieved with a long-pass filter (λ_T _>750 nm, LX902 AcryFilter IR; Mitsubishi Rayon, Tokyo, Japan), which lets infrared light through but filters out all visible light. For the analysis of photomovement, the observation chamber was illuminated from the direction that was being tested (Figures 3a and 3b) using a continuous-output 500-W Xenon short-arc lamp (Ushio Electric, Tokyo, Japan) equipped with an interference filter for microscopic phase contrast illumination (GIF, λ_T (max)_: 540 nm, spectral width [full width at half maximum]: 41 nm; Nikon), which results in green light.

The influence of light intensity on photomovements was analyzed using the 500-W Xenon short-arc lamp and a set of neutral density (ND) filters (ND1, ND3, ND10, ND20, and ND40; Hoya Corporation, Tokyo, Japan). Eight different light intensities (0.00017 μmol m^-2 ^s^-1^, 0.0017 μmol m^-2 ^s^-1^, 0.017 μmol m^-2 ^s^-1^, 0.16 μmol m^-2 ^s^-1^, 2.18 μmol m^-2 ^s^-1^, 19.59 μmol m^-2 ^s^-1^, 191 μmol m^-2 ^s^-1 ^and 457 μmol m^-2 ^s^-1^) were used in the experiments with the vertically oriented observation chamber, and 17 different light intensities (0.00052 μmol m^-2 ^s^-1^, 0.00156 μmol m^-2 ^s^-1^, 0.0052 μmol m^-2 ^s^-1^, 0.0156 μmol m^-2 ^s^-1^,0.052 μmol m^-2 ^s^-1^, 0.156 μmol m^-2 ^s^-1^, 0.52 μmol m^-2 ^s^-1^, 1.04 μmol m^-2 ^s^-1^, 1.56 μmol m^-2 ^s^-1^, 2.08 μmol m^-2 ^s^-1^, 5.2 μmol m^-2 ^s^-1^, 10.4 μmol m^-2 ^s^-1^, 15.6 μmol m^-2 ^s^-1^, 20.8 μmol m^-2 ^s^-1^, 52 μmol m^-2 ^s^-1^, 208 μmol m^-2 ^s^-1 ^and 520 μmol m^-2 ^s^-1^) were used in the experiments with the horizontally oriented observation chamber.

Phototactic movements were quantified by calculating the PI using the formula PI = (B - A) × 100/(100 - A), in which A is the percentage of spheroids located in the investigated area (e.g., area 1) at the moment when the light was turned on (0 s) and B is the percentage of spheroids located in the same area at the respective time after the light was turned on. A high PI reflects a strong photoaccumulation within the given area.

Where applicable, individual images of recorded photomovements were analyzed with respect to the relative distribution of the spheroids in the chamber, the direction of the movements and the velocity of the movements using the NIH Image version 1.62 software [106].

### Recording of fluid streamlines

The fluid streamlines generated by flagella were visualized and recorded according to the method of Hand and Haupt [53], with minor modifications. Briefly, ~40 μl of a suspension of individual *Volvox *spheroids in VT medium was pipetted onto a glass slide, and then 1-5 μl of an aqueous suspension of polystyrene beads (1-μm diameter; Polysciences, Warrington, PA, USA) were added. Two layers of standard vinyl tape were used as spacers between the slide and the coverglass to avoid crushing the spheroids, and the samples were analyzed on an inverted TMS-F microscope (Nikon). Infrared light was used for dark-field illumination, and green light was used for the stimulation of photomovements as described above. The tracks of the polystyrene beads were recorded with a CCD camera (C5985; Hamamatsu Photonics, Hamamatsu, Japan). Both the moving direction and speed of the fluid streamlines were calculated from the tracks of the polystyrene beads on the images.

### Construction and use of a rotating filter system for pulsed light stimulation

A rotating filter system for pulsed light stimulation was constructed on the basis of the data of Foster and Smyth [28,75] using three 2-mm thick acrylic filters, F1 (#700; Sumitomo Chemical, Tokyo, Japan), F2 (#700, Sumitomo Chemical), and F3 (#702; Sumitomo Chemical), with transmissivities for green light of 74.4%, 74.4%, and 40.77%, respectively. Sectors of 28° (F1), 50° (F2) and 110° (F3) were cut out from the filters as calculated in Figure 7e, and the sandwich construction was mounted reflection-symmetrically on a DC servomotor as shown in Figure 7b. The sandwich construction resulted in four different transmissivities: T1 = 100% (gap, no filter); T2 = 74% (F1), T3 = 55% (F1 + F2), and T4 = 23% (F1 + F2 + F3). The filter system was rotated at 0.83 rps (49.8 rpm). The rotating filter system was placed into the light path of the green light that was used to stimulate the photomovements. The oscillating light intensity was recorded by a silicon photodiode sensor (S1336-8BQ; Hamamatsu Photonics) connected to an oscilloscope (Tektronix, Beaverton, OR); the highest recorded light intensity (28 μmol m^-2 ^s^-1^) was set to 100%.

### Analysis of flagellar waveforms

Flagellar waveforms were analyzed both with the CCD camera on the TMS-F microscope as described above using a long-pass filter with λ_T _>610 nm for field illumination, and by high-speed microvideography as reported by Inouye and Hori [107]. In the latter case, flagellar activity (at 20°C) was filmed at 200 frames per second using an MHS-200 high-speed video system (NAC Image Technology, Simi Valley, CA) attached to an Optiphoto light microscope (Nikon). The built-in strobe light of the high-speed video system was used to illuminate the field of view. Because the strobe light is indispensable for high-speed microvideography, the normal beating mode was recorded either right at the moment when the dark-adapted spheroids were illuminated with the strobe light, which corresponded to beating in the dark as the cells had no time to react, or after 10 s of illumination with the strobe light, which was several seconds after cells had adapted to the light.

### Analysis of eyespot sizes

The spheroids were flattened gently by placing a suspension of algae between a slide (without vinyl tape spacers) and a coverglass. The flattened spheroids were observed with bright-field illumination using an upright compound microscope (BHS; Olympus, Tokyo, Japan). The digital images were analyzed using NIH Image software to calculate the eyespot size, defined as the largest diameter of the eyespots, and to determine the linear distance between a given eyespot and the anterior pole [106]. The anterior pole of the spheroid was identified as described earlier [5].

## Abbreviations

F: filter; n: sample size; PI: photoaccumulation index; SD: standard deviation; T: transmissivity.

## Authors' contributions

NU performed experiments and analyzed data. SM designed experiments, performed experiments, and contributed reagents and materials. II designed experiments and coordinated the first phase of the research project. AH (corresponding author) evaluated the data, coordinated the second phase of the research project, constructed the figures and wrote the paper. All of the authors read and approved the final manuscript.

## Supplementary Material

Additional file 1 **Phylogenetic relationship between *Volvox rousseletii *MI01 and other volvocine algae**. This analysis is based on a combined data set of the *psa*A, *psa*B, and *rbc*L cDNA fragments from 47 volvocine species and/or strains. The unrooted tree was calculated by the neighbor-joining method using the PHYLIP software. Click here for file

Additional file 2 **Description of the phylogenetic analysis of the *Volvox rousseletii *strain MI01 that was utilized in this study**. The *Volvox rousseletii *strain MI01 utilized in this study was subjected to a molecular phylogenetic analysis. To do so, certain DNA fragments that were used in the phylogenetic analyses of other volvocine algae were cloned and sequenced, which included the chloroplast genes encoding the photosystem I P700 chlorophyll a apoprotein A1 (*psa*A), the photosystem I P700 chlorophyll a apoprotein A2 (*psa*B), ribulose bisphosphate carboxylase (*rbc*L) and the internal transcribed spacer sequence 2 (ITS2). Click here for file

Additional file 3 **List of species, strains, abbreviations, accession numbers, and references for the *psa*A, *psa*B, *rbc*L and ITS2 sequences from volvocine species**. Click here for file

Additional file 4 **Sequence alignment of *psa*A cDNA fragments from several volvocine species**. Click here for file

Additional file 5 **Sequence alignment of *psa*B cDNA fragments from several volvocine species**. Click here for file

Additional file 6 **Sequence alignment of *rbc*L cDNA fragments from several volvocine species**. Click here for file

Additional file 7 **Comparison of *psa*A sequences from several volvocine species**. Click here for file

Additional file 8 **Comparison of *psa*B sequences from several volvocine species**. Click here for file

Additional file 9 **Comparison of *rbc*L sequences from several volvocine species**. Click here for file

Additional file 10 **Comparison of ITS2 sequences from several volvocine species**. Click here for file

## References

[B1] Szathmáry E Smith JM The major evolutionary transitions Nature 1995 374 227 232 10.1038/374227a0 7885442

[B2] Bonner JT The origins of multicellularity Integr Biol 1998 1 27 36 10.1002/(SICI)1520-6602(1998)1:1<27::AID-INBI4>3.0.CO;2-6

[B3] Grosberg RK Strathmann R The evolution of multicellularity: a minor major transition? Annu Rev Ecol Evol Syst 2007 38 621 654 10.1146/annurev.ecolsys.36.102403.114735

[B4] Prochnik SE Umen J Nedelcu A Hallmann A Miller SM Nishii I Ferris P Kuo A Mitros T Fritz-Laylin LK Hellsten U Chapman J Simakov O Rensing SA Terry A Pangilinan J Kapitonov V Jurka J Salamov A Shapiro H Schmutz J Grimwood J Lindquist E Lucas S Grigoriev IV Schmitt R Kirk D Rokhsar DS Genomic analysis of organismal complexity in the multicellular green alga *Volvox carteri* Science 2010 329 223 226 10.1126/science.1188800 20616280 PMC2993248

[B5] Kirk DL Volvox: molecular-genetic origins of multicellularity and cellular differentiation 1998

[B6] Kirk DL A twelve-step program for evolving multicellularity and a division of labor BioEssays 2005 27 299 310 10.1002/bies.20197 15714559

[B7] Herron MD Hackett JD Aylward FO Michod RE Triassic origin and early radiation of multicellular volvocine algae Proc Natl Acad Sci USA 2009 106 3254 3258 10.1073/pnas.0811205106 19223580 PMC2651347

[B8] Hoops HJ Somatic cell flagellar apparatuses in two species of *Volvox *(Chlorophyceae) J Phycol 1984 20 20 27 10.1111/j.0022-3646.1984.00020.x

[B9] Larson A Kirk MM Kirk DL Molecular phylogeny of the volvocine flagellates Mol Biol Evol 1992 9 85 105 1552843 10.1093/oxfordjournals.molbev.a040710

[B10] Nozaki H Itoh M Sano R Uchida H Watanabe MM Kuroiwa T Phylogenetic relationships within the colonial Volvocales (Chlorophyta) inferred from *rbc*L gene sequence data J Phycol 1995 31 970 979 10.1111/j.0022-3646.1995.00970.x

[B11] Coleman AW Phylogenetic analysis of "Volvocaceae" for comparative genetic studies Proc Natl Acad Sci USA 1999 96 13892 13897 10.1073/pnas.96.24.13892 10570169 PMC24161

[B12] Nozaki H Ohta N Takano H Watanabe MM Reexamination of phylogenetic relationships within the colonial Volvocales (Chlorophyta): an analysis of *atpB *and *rbcL *gene sequences J Phycol 1999 35 104 112 10.1046/j.1529-8817.1999.3510104.x

[B13] Nozaki H Takahara M Nakazawa A Kita Y Yamada T Takano H Kawano S Kato M Evolution of *rbc*L group IA introns and intron open reading frames within the colonial Volvocales (Chlorophyceae) Mol Phylogenet Evol 2002 23 326 338 10.1016/S1055-7903(02)00030-1 12099791

[B14] Smith GM A comparative study of the species of *Volvox* Trans Am Microsc Soc 1944 63 265 310 10.2307/3223302

[B15] Nozaki H Ito M Sano R Uchida H Watanabe MM Takahashi H Kuroiwa T Phylogenetic analysis of *Yamagishiella *and *Platydorina *(Volvocaceae, Chlorophyta) based on *rbc*L gene sequences J Phycol 1997 33 272 278 10.1111/j.0022-3646.1997.00272.x

[B16] Nozaki H Origin and evolution of the genera *Pleodorina *and *Volvox *(Volvocales) Biologia (Bratisl) 2003 58 425 431

[B17] Nozaki H Ott FD Coleman AW Morphology, molecular phylogeny and taxonomy of two new species of *Pleodorina *(Volvoceae, Chlorophyceae) J Phycol 2006 42 1072 1080 10.1111/j.1529-8817.2006.00255.x

[B18] Häder DP Lebert M Photoorientation in photosynthetic flagellates Methods Mol Biol 2009 571 51 65 10.1007/978-1-60761-198-1_3 19763958

[B19] Nagel G Ollig D Fuhrmann M Kateriya S Musti AM Bamberg E Hegemann P Channelrhodopsin-1: a light-gated proton channel in green algae Science 2002 296 2395 2398 10.1126/science.1072068 12089443

[B20] Sineshchekov OA Jung KH Spudich JL Two rhodopsins mediate phototaxis to low- and high-intensity light in *Chlamydomonas reinhardtii* Proc Natl Acad Sci USA 2002 99 8689 8694 12060707 10.1073/pnas.122243399 PMC124360

[B21] Suzuki T Yamasaki K Fujita S Oda K Iseki M Yoshida K Watanabe M Daiyasu H Toh H Asamizu E Tabata S Miura K Fukuzawa H Nakamura S Takahashi T Archaeal-type rhodopsins in *Chlamydomonas*: model structure and intracellular localization Biochem Biophys Res Commun 2003 301 711 717 10.1016/S0006-291X(02)03079-6 12565839

[B22] Nagel G Szellas T Huhn W Kateriya S Adeishvili N Berthold P Ollig D Hegemann P Bamberg E Channelrhodopsin-2, a directly light-gated cation-selective membrane channel Proc Natl Acad Sci USA 2003 100 13940 13945 10.1073/pnas.1936192100 14615590 PMC283525

[B23] Zhang F Prigge M Beyriere F Tsunoda SP Mattis J Yizhar O Hegemann P Deisseroth K Red-shifted optogenetic excitation: a tool for fast neural control derived from *Volvox carteri* Nat Neurosci 2008 11 631 633 10.1038/nn.2120 18432196 PMC2692303

[B24] Kianianmomeni A Stehfest K Nematollahi G Hegemann P Hallmann A Channelrhodopsins of *Volvox carteri *are photochromic proteins that are specifically expressed in somatic cells under control of light, temperature, and the sex inducer Plant Physiol 2009 151 347 366 10.1104/pp.109.143297 19641026 PMC2736010

[B25] Dieckmann CL Eyespot placement and assembly in the green alga *Chlamydomonas* BioEssays 2003 25 410 416 10.1002/bies.10259 12655648

[B26] Schmidt M Gessner G Luff M Heiland I Wagner V Kaminski M Geimer S Eitzinger N Reissenweber T Voytsekh O Fiedler M Mittag M Kreimer G Proteomic analysis of the eyespot of *Chlamydomonas reinhardtii *provides novel insights into its components and tactic movements Plant Cell 2006 18 1908 1930 10.1105/tpc.106.041749 16798888 PMC1533972

[B27] Hegemann P Algal sensory photoreceptors Annu Rev Plant Biol 2008 59 167 189 10.1146/annurev.arplant.59.032607.092847 18444900

[B28] Kreimer G The green algal eyespot apparatus: a primordial visual system and more? Curr Genet 2009 55 19 43 10.1007/s00294-008-0224-8 19107486

[B29] Hoops HJ Motility in the colonial and multicellular Volvocales: structure, function, and evolution Protoplasma 1997 199 99 112 10.1007/BF01294499

[B30] Ringo DL Flagellar motion and fine structure of the flagellar apparatus in *Chlamydomonas* J Cell Biol 1967 33 543 571 10.1083/jcb.33.3.543 5341020 PMC2107204

[B31] Feinleib MEH Curry GM The relationship between stimulus intensity and oriented phototactic response (topotaxis) in *Chlamydomonas* Physiol Plant 1971 25 346 352 10.1111/j.1399-3054.1971.tb01453.x

[B32] Rüffer U Nultsch W High-speed cinematographic analysis of the movement of *Chlamydomonas* Cell Motil 1985 5 251 263 10.1002/cm.970050307

[B33] Rüffer U Nultsch W Comparison of the beating of *cis*- and *trans*-flagella of *Chlamydomonas *cells held on micropipettes Cell Motil Cytoskeleton 1987 7 87 93 10.1002/cm.970070111 9858155

[B34] Rüffer U Nultsch W Flagellar photoresponses of *Chlamydomonas *cells held on micropipettes: I. Change in flagellar beat frequency Cell Motil Cytoskeleton 1990 15 162 167 10.1002/cm.970150305

[B35] Rüffer U Nultsch W Flagellar photoresponses of *Chlamydomonas *cells held on micropipettes: II. Change in flagellar beat pattern Cell Motil Cytoskeleton 1991 18 269 278 10.1002/cm.970180404

[B36] Polin M Tuval I Drescher K Gollub JP Goldstein RE *Chlamydomonas *swims with two "gears" in a eukaryotic version of run-and-tumble locomotion Science 2009 325 487 490 10.1126/science.1172667 19628868

[B37] Takahashi T Yoshihara K Watanabe M Kubota M Johnson R Derguini F Nakanishi K Photoisomerization of retinal at 13-ene is important for phototaxis of *Chlamydomonas reinhardtii*: simultaneous measurements of phototactic and photophobic responses Biochem Biophys Res Commun 1991 178 1273 1279 10.1016/0006-291X(91)91031-7 1872847

[B38] Rüffer U Nultsch W Flagellar coordination in *Chlamydomonas *cells held on micropipettes Cell Motil Cytoskeleton 1998 41 297 307 9858155 10.1002/(SICI)1097-0169(1998)41:4<297::AID-CM3>3.0.CO;2-Y

[B39] Hyams JS Borisy GG Isolated flagellar apparatus of *Chlamydomonas*: characterization of forward swimming and alteration of waveform and reversal of motion by calcium ions in vitro J Cell Sci 1978 33 235 253 31367 10.1242/jcs.33.1.235

[B40] Bessen M Fay RB Witman GB Calcium control of waveform in isolated flagellar axonemes of *Chlamydomonas* J Cell Biol 1980 86 446 455 10.1083/jcb.86.2.446 6447155 PMC2111489

[B41] Witman GB *Chlamydomonas *phototaxis Trends Cell Biol 1993 3 403 408 10.1016/0962-8924(93)90091-E 14731659

[B42] Gerisch G Die Zelldifferenzierung bei *Pleodorina californica *Shaw und die Organisation der Phytomonadinenkolonien Arch Protistenkd 1959 104 292 358

[B43] Hoops HJ Floyd GL Ultrastructure and development of the flagellar apparatus and flagellar motion in the colonial green alga *Astrephomene gubernaculifera* J Cell Sci 1983 63 21 41 6630310 10.1242/jcs.63.1.21

[B44] Hoops HJ Flagellar, cellular and organismal polarity in *Volvox carteri* J Cell Sci 1993 104 105 117

[B45] Greuel BT Floyd GL Development of the flagellar apparatus and flagellar orientation in the colonial green alga *Gonium pectorale *(Volvocales) J Phycol 1985 21 358 371

[B46] Mast SO Reactions to light in *Volvox*, with special reference to the process of orientation J Comp Physiol A Neuroethol Sens Neural Behav Physiol 1926 4 637 658

[B47] Linnaeus C Systema naturae. Regnum animale Stockholm 1758 10

[B48] Holmes SJ Phototaxis in *Volvox* Biol Bull 1903 4 319 326 10.2307/1535852

[B49] Mast SO Light reactions in lower organisms. II. *Volvox globator* J Comp Neurol Psychol 1907 17 99 180 10.1002/cne.920170202

[B50] Mast SO Light and the behavior of organisms 1911 1 New York: J. Wiley & Sons

[B51] Mast SO The process of orientation in the colonial organism, *Gonium pectorale*, and a study of the structure and function of the eye-spot J Exp Zool 1916 20 1 17 10.1002/jez.1400200102

[B52] Huth K Bewegung und Orientierung bei *Volvox aureus *Ehrb. I. Mechanismus der phototaktischen Reaktion Z Pflanzenphysiol 1970 62 436 450

[B53] Hand WG Haupt W Flagellar activity of the colony members of *Volvox aureus *Ehrbg. during light stimulation J Protozool 1971 18 361 364

[B54] Sakaguchi H Tawada K Temperature effect on the photo-accumulation and phobic response of *Volvox aureus* J Protozool 1977 24 284 288

[B55] Sakaguchi H Iwasa K Two photophobic responses in *Volvox carteri* Plant Cell Physiol 1979 20 909 916

[B56] Sakaguchi H Effect of external ionic environment on phototaxis of *Volvox carteri* Plant Cell Physiol 1979 20 1643 1651

[B57] Hoops HJ Brighton MC Stickles SM Clement PR A test of two possible mechanisms for phototactic steering in *Volvox carteri *(Chlorophyceae) J Phycol 1999 35 539 547 10.1046/j.1529-8817.1999.3530539.x

[B58] Braun FJ Hegemann P Two light-activated conductances in the eye of the green alga *Volvox carteri* Biophys J 1999 76 1668 1678 10.1016/S0006-3495(99)77326-1 10049347 PMC1300143

[B59] Ebnet E Fischer M Deininger W Hegemann P Volvoxrhodopsin, a light-regulated sensory photoreceptor of the spheroidal green alga *Volvox carteri* Plant Cell 1999 11 1473 1484 10.1105/tpc.11.8.1473 10449581 PMC144291

[B60] Solari CA Ganguly S Kessler JO Michod RE Goldstein RE Multicellularity and the functional interdependence of motility and molecular transport Proc Natl Acad Sci USA 2006 103 1353 1358 10.1073/pnas.0503810103 16421211 PMC1360517

[B61] Drescher K Goldstein RE Tuval I Fidelity of adaptive phototaxis Proc Natl Acad Sci USA 2010 107 11171 11176 10.1073/pnas.1000901107 20534560 PMC2895142

[B62] Meyer A Die Plasmaverbindung und die Membranen von *Volvox globator*, *aureus *und *tertius*, mit Rücksicht auf die thierischen Zellen Bot Zeitg 1896 54 187 217

[B63] Hoops HJ Nishii I Kirk DL Baluska F, Volkmann D, Barlow PW Cytoplasmic bridges in *Volvox *and its relatives Cell-Cell Channels 2005 Georgetown, Texas: Eurekah.com 1 20

[B64] Schletz K Phototaxis bei *Volvox*. Pigmentsysteme der Lichtrichtungsperzeption Z Pflanzenphysiol 1976 77 189 211

[B65] Fritsch FE The structure and reproduction of the algae. Vol. 1: Introduction, Chlorophyceae, Xanthophyceae, Chrysophyceae, Bacillariophyceae, Cryptophyceae, Dinophyceae, Chloromonadineae, Euglenineae, colourless flagellata 1935 Cambridge, UK: Cambridge University Press

[B66] Coleman AW Suarez A Goff LJ Molecular delineation of species and syngens in volvocacean green algae (Chlorophyta) J Phycol 1994 30 80 90 10.1111/j.0022-3646.1994.00080.x

[B67] Mai JC Coleman AW The internal transcribed spacer 2 exhibits a common secondary structure in green algae and flowering plants J Mol Evol 1997 44 258 271 10.1007/PL00006143 9060392

[B68] Coleman AW Mai JC Ribosomal DNA ITS-1 and ITS-2 sequence comparisons as a tool for predicting genetic relatedness J Mol Evol 1997 45 168 177 10.1007/PL00006217 9236277

[B69] Coleman AW Preparata RM Mehrotra B Mai JC Derivation of the secondary structure of the ITS-1 transcript in Volvocales and its taxonomic correlations Protist 1998 149 135 146 10.1016/S1434-4610(98)70018-5 23196163

[B70] Nozaki H Misawa K Kajita T Kato M Nohara S Watanabe MM Origin and evolution of the colonial Volvocales (Chlorophyceae) as inferred from multiple, chloroplast gene sequences Mol Phylogenet Evol 2000 17 256 268 10.1006/mpev.2000.0831 11083939

[B71] Pedley TJ Kessler JO Hydrodynamic phenomena in suspensions of swimming microorganisms Annu Rev Fluid Mech 1992 24 313 358 10.1146/annurev.fl.24.010192.001525

[B72] Drescher K Leptos KC Tuval I Ishikawa T Pedley TJ Goldstein RE Dancing *Volvox*: Hydrodynamic bound states of swimming algae Phys Rev Lett 2009 102 168101 10.1103/PhysRevLett.102.168101 19518757 PMC4833199

[B73] Solari CA Michod RE Goldstein RE *Volvox barberi*, the fastest swimmer of the Volvocales (Chlorophyceae) J Phycol 2008 44 1395 1398 10.1111/j.1529-8817.2008.00603.x 27039854

[B74] Solari CA Kessler JO Michod RE A hydrodynamics approach to the evolution of multicellularity: flagellar motility and germ-soma differentiation in volvocalean green algae Am Nat 2006 167 537 554 10.1086/501031 16670996

[B75] Foster KW Smyth RD Light antennas in phototactic algae Microbiol Rev 1980 44 572 630 7010112 10.1128/mr.44.4.572-630.1980 PMC373196

[B76] Nakamura S Tamm SL Calcium control of ciliary reversal in ionophore-treated and ATP-reactivated comb plates of ctenophores J Cell Biol 1985 100 1447 1454 10.1083/jcb.100.5.1447 3921553 PMC2113888

[B77] Tamm SL Terasaki M Visualization of calcium transients controlling orientation of ciliary beat J Cell Biol 1994 125 1127 1135 10.1083/jcb.125.5.1127 8195294 PMC2120050

[B78] Mackie GO Spencer AN Strathmann R Electrical activity associated with ciliary reversal in an echinoderm larva Nature 1969 223 1384 1385 10.1038/2231384a0

[B79] Galt CP Mackie GO Electrical correlates of ciliary reversal in *Oikopleura* J Exp Biol 1971 55 205 212

[B80] Naitoh Y Eckert R Sleigh MA The control of ciliary activity in protozoa Cilia and flagella 1974 London: Academic Press 305 352

[B81] Naitoh Y Eckert R Ionic mechanisms controlling behavioral responses of *Paramecium *to mechanical stimulation Science 1969 164 963 965 10.1126/science.164.3882.963 5768366

[B82] Naitoh Y Eckert R Friedman K A regenerative calcium response in *Paramecium* J Exp Biol 1972 56 667 681 4668720 10.1242/jeb.56.3.667

[B83] Nakaoka Y Ooi H Regulation of ciliary reversal in triton-extracted *Paramecium *by calcium and cyclic adenosine monophosphate J Cell Sci 1985 77 185 195 3003129 10.1242/jcs.77.1.185

[B84] Naitoh Y Kaneko H Reactivated triton-extracted models of *Paramecium*: modification of ciliary movement by calcium ions Science 1972 176 523 524 10.1126/science.176.4034.523 5032354

[B85] Stavis RL Hirschberg R Phototaxis in *Chlamydomonas reinhardtii* J Cell Biol 1973 59 367 377 10.1083/jcb.59.2.367 4805005 PMC2109087

[B86] Stavis RL The effect of azide on phototaxis in *Chlamydomonas reinhardi* Proc Natl Acad Sci USA 1974 71 1824 1827 10.1073/pnas.71.5.1824 4525466 PMC388334

[B87] Schmidt JA Eckert R Calcium couples flagellar reversal to photostimulation in *Chlamydomonas reinhardtii* Nature 1976 262 713 715 10.1038/262713a0 958445

[B88] Litvin FF Sineshchekov OA Sineshchekov VA Photoreceptor electric potential in the phototaxis of the alga *Haematococcus pluvialis* Nature 1978 271 476 478 10.1038/271476a0 628427

[B89] Nultsch W Effect of external factors on phototaxis of *Chlamydomonas reinhardtii*. III. Cations Arch Microbiol 1979 123 93 99 10.1007/BF00403506 1156091

[B90] Kamiya R Witman GB Submicromolar levels of calcium control the balance of beating between the two flagella in demembranated models of *Chlamydomonas* J Cell Biol 1984 98 97 107 10.1083/jcb.98.1.97 6707098 PMC2112995

[B91] Omoto CK Brokaw CJ Bending patterns of *Chlamydomonas *flagella: II. Calcium effects on reactivated *Chlamydomonas *flagella Cell Motil 1985 5 53 60 10.1002/cm.970050105 3978704

[B92] Harz H Hegemann P Rhodopsin-regulated calcium currents in *Chlamydomonas* Nature 1991 351 489 491 10.1038/351489a0

[B93] Horst CJ Witman GB ptx1, a nonphototactic mutant of *Chlamydomonas*, lacks control of flagellar dominance J Cell Biol 1993 120 733 741 10.1083/jcb.120.3.733 8425899 PMC2119553

[B94] Beck C Uhl R On the localization of voltage-sensitive calcium channels in the flagella of *Chlamydomonas reinhardtii* J Cell Biol 1994 125 1119 1125 10.1083/jcb.125.5.1119 8195293 PMC2120057

[B95] Pazour GJ Sineshchekov OA Witman GB Mutational analysis of the phototransduction pathway of *Chlamydomonas reinhardtii* J Cell Biol 1995 131 427 440 10.1083/jcb.131.2.427 7593169 PMC2199980

[B96] Yoshimura K Shingyoji C Takahashi K Conversion of beating mode in *Chlamydomonas *flagella induced by electric stimulation Cell Motil Cytoskeleton 1997 36 236 245 9067619 10.1002/(SICI)1097-0169(1997)36:3<236::AID-CM4>3.0.CO;2-5

[B97] Okita N Isogai N Hirono M Kamiya R Yoshimura K Phototactic activity in *Chlamydomonas *'non-phototactic' mutants deficient in Ca^2+^-dependent control of flagellar dominance or in inner-arm dynein J Cell Sci 2005 118 529 537 10.1242/jcs.01633 15657081

[B98] Kam V Moseyko N Nemson J Feldman LJ Gravitaxis in *Chlamydomonas reinhardtii*: characterization using video microscopy and computer analysis Int J Plant Sci 1999 160 1093 1098 10.1086/314205 10568776

[B99] Yoshimura K Matsuo Y Kamiya R Gravitaxis in *Chlamydomonas reinhardtii *studied with novel mutants Plant Cell Physiol 2003 44 1112 1118 10.1093/pcp/pcg134 14581636

[B100] Kessler JO The external dynamics of swimming microorganisms Prog Phycol Res 1986 4 257 307

[B101] Huth K Bewegung und Orientierung bei *Volvox aureus *Ehrb. II. Richtungsabweichung bei taktischen Reaktionen Z Pflanzenphysiol 1970 63 344 351

[B102] Provasoli L Pintner IJ Tryon CA, Hartman RT Artificial media for fresh-water algae: problems and suggestions The Ecology of Algae, a symposium held at the Pymatuning Laboratory of Field Biology on June 18 and 19, 1959 1959 1 Pittsburgh, PA: The Pymatuning Symposia in Ecology, Special Publication No. 2., University of Pittsburgh 84 96

[B103] Watanabe MM Nozaki H NIES-collection: List of strains. Microalgae and protozoa 1994 4 Tsukuba, Japan: National Institute for Environmental Studies

[B104] Nozaki H Kuroiwa H Mita T Kuroiwa T *Pleodorina japonica *sp. nov. (Volvocales, Chlorophyta) with bacteria-like endosymbionts Phycologia 1989 28 252 267

[B105] Kasai F Kawachi M Erata M Watanabe MM NIES-collection: List of strains. Microalgae and protozoa 2004 7 Tsukuba, Japan: National Institute for Environmental Studies

[B106] Rasband WS Bright DS NIH Image: a public domain image processing program for the Macintosh Microbeam Anal Soc J 1995 4 137 149

[B107] Inouye I Hori T High-speed video analysis of the flagellar beat and swimming patterns of algae: possible evolutionary trends in green algae Protoplasma 1991 164 54 59 10.1007/BF01320815

