# Supplementary material for: How 5000 independent rowers coordinate their strokes in order to row into the sunlight: Phototaxis in the multicellular green alga Volvox
Source: BMC Biol. 2010 Jul 27;8:103. doi: 10.1186/1741-7007-8-103 (PMC2920248; doi:10.1186/1741-7007-8-103)
Supplement: Additional file 1 — Phylogenetic relationship between Volvox rousseletii MI01 and other volvocine algae. This analysis is based on a combined data set of the psaA, psaB, and rbcL cDNA fragments from 47 volvocine species and/or strains. The unrooted tree was calculated by the neighbor-joining method using the PHYLIP software. [file 1741-7007-8-103-S1.PDF]

Phylogenetic relationship between *Volvox rousseletii* MI01 and other volvocine algae

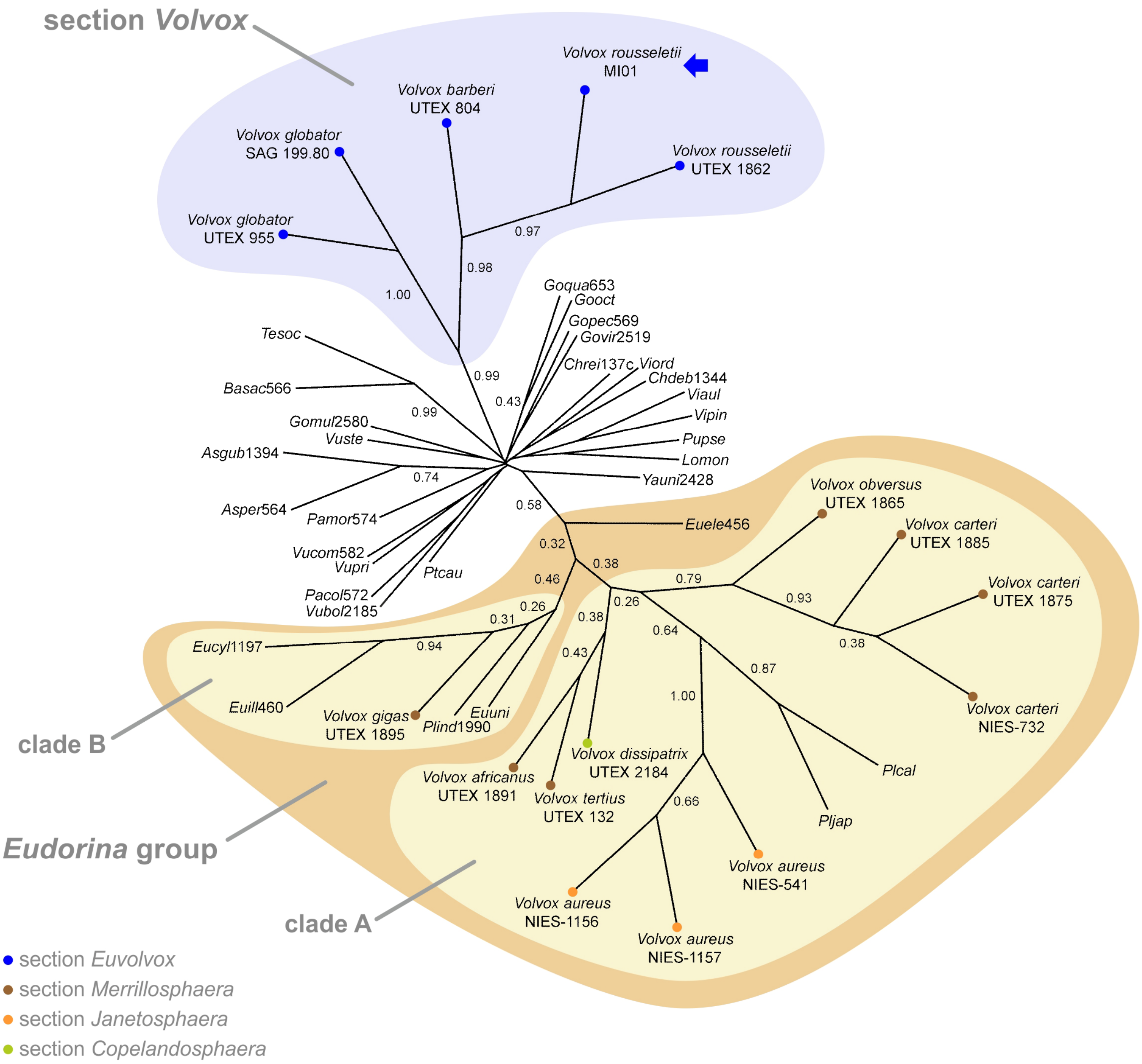

This analysis is based on a combined data set of the *psa*A, *psa*B, and *rbc*L cDNA fragments from 47 volvocine and/or strains. The term “volvocine species/strains” refers to a group of species closely related to the “genus” *Volvox* within the order Volvocales (Chlorophyta); this group spans the full range of complexity from unicellular genera (like *Chlamydomonas*), via colonial genera (like *Eudorina*), to multicellular genera (like *Volvox*) (Kirk, 1998). The unrooted

tree was calculated by the neighbor-joining method (Saitou and Nei, 1987) of the PHYLIP software (Felsenstein, 1989). The numbers indicate bootstrap analysis values obtained using 30,000 resampled data sets. Low values were omitted; short branches reflect little support. The analysis is based on the alignments given in Additional Files 4 (*psa*A), 5 (*psa*B), and 6 (*rbc*L).

In a study of Smith (1944), the "genus" *Volvox* was subdivided into four sections on the basis of differences in vegetative morphology: the section *Merrillosphaera*, the section *Janetosphaera*, the section *Copelandosphaera* and the section *Euvolvox*. Species in the section *Merrillosphaera* (e.g. *V. carteri*) have no cytoplasmic bridges in adults, cells have a round shape and the cellular compartments of the extracellular matrix (ECM) are complete; species in the section *Euvolvox* (e.g. *V. rousseletii*) have broad cytoplasmic bridges in adults, cells appear stellate when viewed from their flagellar ends and the cellular compartments of the ECM are complete; species in the section *Janetosphaera* (e.g. *V. aureus*) have moderate cytoplasmic bridges in adults, cells have a round shape and the cellular compartments of the ECM lack walls and floors; there is only a single species in the section *Copelandosphaera* (*V. dissipatrix*), which has very fine cytoplasmic bridges in adults, cells have a round shape and the cellular compartments of the ECM lack floors (Smith, 1944; Kirk, 1998). The term "section *Euvolvox*" was later replaced by "section *Volvox*". The affiliations of species of the "genus" *Volvox* to a particular section according to Smith (1944) are indicated in our phylogenetic tree by colored dots.

More recent molecular phylogenetic analyses revealed a polyphyletic origin of the "genus" *Volvox* (Coleman, 1999; Nozaki et al., 1999; Nozaki, 2003; Nozaki et al., 2006). After reexamination of phylogenetic relationships, species of the former sections *Merrillosphaera*, *Janetosphaera* and *Copelandosphaera* were positioned within a new "*Eudorina* group" (Coleman, 1999; Nozaki et al., 1999; Nozaki, 2003; Nozaki et al., 2006); the *Eudorina* group contains two major clades, A and B (Nozaki et al., 2006). Outside the *Eudorina* group, three species of the "genus" *Volvox*, *V. rousseletii*, *V. globator* and *V. barberi*, formed a robust monophyletic group, the section *Volvox*, which is identical with the former section *Euvolvox*. Our present analysis and the phylogentic tree shown above are consistent with these previous molecular analyses. The affiliations of species to a particular group, section or clade (according to Nozaki et al., 2006) are indicated in our phylogenetic tree by colored areas.

The position of *Volvox rousseletii* MI01, which was used in this study, is indicated by a blue arrow. The names of all species of the "genus" *Volvox* are spelled out. Abbreviations of other species/strains are explained in Additional File 3.

References

- Coleman AW: **Phylogenetic analysis of "Volvocaceae" for comparative genetic studies**. Proc Natl Acad Sci USA 1999, **96**:13892-13897.

- Felsenstein J: **Phylip - Phylogeny Inference Package (Version 3.2)**. *Cladistics* 1989, **5**:164-166.

- Kirk DL: ***Volvox*: molecular-genetic origins of multicellularity and cellular differentiation**. Cambridge: Cambridge University Press; 1998.

- Nozaki H: **Origin and evolution of the genera *Pleodorina* and *Volvox* (Volvocales)**. Biologia (Bratisl) 2003, **58**:425-431.

- Nozaki H, Ohta N, Takano H, Watanabe MM: **Reexamination of phylogenetic relationships within the colonial Volvocales (Chlorophyta): an analysis of *atp*B and *rbc*L gene sequences**. J Phycol 1999, **35**:104-112.

- Nozaki H, Ott FD, Coleman AW: **Morphology, molecular phylogeny and taxonomy of two new species of *Pleodorina* (Volvoceae, Chlorophyceae)**. J Phycol 2006, **42**:1072-1080.

- Saitou N, Nei M: **The neighbor-joining method: a new method for reconstructing phylogenetic trees**. *Mol Biol Evol* 1987, **4**:406-425.

- Smith GM: **A comparative study of the species of *Volvox***. Trans Am Microsc Soc 1944, **63**:265-310.
