# Supplementary material for: How 5000 independent rowers coordinate their strokes in order to row into the sunlight: Phototaxis in the multicellular green alga Volvox
Source: BMC Biol. 2010 Jul 27;8:103. doi: 10.1186/1741-7007-8-103 (PMC2920248; doi:10.1186/1741-7007-8-103)
Supplement: Additional file 2 — Description of the phylogenetic analysis of the Volvox rousseletii strain MI01 that was utilized in this study. The Volvox rousseletii strain MI01 utilized in this study was subjected to a molecular phylogenetic analysis. To do so, certain DNA fragments that were used in the phylogenetic analyses of other volvocine algae were cloned and sequenced, which included the chloroplast genes encoding the photosystem I P700 chlorophyll a apoprotein A1 (psaA), the photosystem I P700 chlorophyll a apoprotein A2 (psaB), ribulose bisphosphate carboxylase (rbcL) and the internal transcribed spacer sequence 2 (ITS2). [file 1741-7007-8-103-S2.PDF]

Description of the phylogenetic analysis of the *Volvox rousseletii* strain MI01 that was utilized in this study

Our standard *Volvox rousseletii* strain is MI01, which we use since several years. This strain was originally isolated from the Machi-ike pond in Oh-gata, Tsukuba-shi, Ibaraki, Japan in 1998. We checked the identity of MI01 by cloning and sequencing of certain DNA fragments that have been used in phylogenetic analyses of other volvocine algae (Nozaki et al., 2000; Nozaki, 2003; Coleman et al., 1994; Mai and Coleman, 1997; Coleman and Mai, 1997; Coleman et al., 1998; Coleman, 1999). The term “volvocine species/strains” refers to a group of species closely related to the “genus” *Volvox* within the order Volvocales (Chlorophyta); this group spans the full range of complexity from unicellular genera (like *Chlamydomonas*), via colonial genera (like *Eudorina*), to multicellular genera (like *Volvox*) (Kirk, 1998). The analyzed sequences from volvocine algae included fragments of chloroplast genes encoding photosystem I P700 chlorophyll a apoprotein A1 (*psa*A), photosystem I P700 chlorophyll a apoprotein A2 (*psa*B) and ribulose bisphosphate carboxylase (*rbc*L) (Nozaki et al., 2000; Nozaki, 2003), as well as the internal transcribed spacer sequence 2 (ITS2) (Coleman et al., 1994; Mai and Coleman, 1997; Coleman and Mai, 1997; Coleman et al., 1998; Coleman, 1999). The used species, strains, abbreviations, accession numbers, and references for *psa*A, *psa*B, *rbc*L and ITS2 sequences from volvocine species are listed in Additional File 3. Oligonucleotide primers for PCR were designed based on multiple alignments of known sequences from other volvocine species/strains. The sequences of the obtained DNA fragments from *Volvox rousseletii* strain MI01 were aligned with previously known sequences from volvocine species/strains, and the alignments are shown in Additional Files 4 (*psa*A), 5 (*psa*B), and 6 (*rbc*L). It was not possible to make a reasonable alignment with the investigated ITS2 sequences due to weak or missing similarities between several sequences. Sequence identities with several related sequences from other volvocine species/strains were also calculated individually and are shown in Additional Files 7 (*psa*A), 8 (*psa*B), and 9 (*rbc*L). ITS2 sequences also were compared in this way and the results are shown in Additional File 10.

As expected, *Volvox rousseletii* strain MI01 always shows the highest sequence identities with *Volvox rousseletii* strain UTEX 1862 followed by high identities with other members of the section *Volvox*. Both *V. rousseletii* strains match perfectly for *psa*A, *psa*B and *rbc*L and there is 97% identity for ITS2.

Based on the sequence alignments shown in Additional Files 4 (*psa*A), 5 (*psa*B), and 6 (*rbc*L), 10,000 phylogenetic trees of volvocine algae have been calculated for each of the three genes (it was not possible to do this for ITS2 sequences due to the weak similarities). A

consensus tree was calculated from the resulting 30,000 trees, which is shown in Additional File 1.

Volvox rousseletii strain MI01 is located next to *Volvox rousseletii* strain UTEX 1862 within the Volvocales section *Volvox*.

Methods

Isolation of genomic DNA

Genomic DNA was prepared from *Volvox* spheroids using a DNeasy Plant Mini Kit (Qiagen, Hilden, Germany). We checked the purity and quantity of the DNA using agarose gel electrophoresis and UV spectrophotometry (Ultrospec 2100 Pro UV/Visible Spectrophotometer; GE Healthcare, Uppsala, Sweden).

Primer design

For all PCR reactions, we designed the oligonucleotide primers using the following primer analysis software packages: Oligo 6 (Molecular Biology Insights, Cascade, CO), DNASIS (version 7.00; Hitachi Software Engineering, South San Francisco, CA), or Primer Express (Applied Biosystems, Foster City, CA).

Genomic PCR

Genomic PCR amplification was carried out in a total volume of 25 µl, which contained ~100 ng of genomic DNA, 300 nM of each primer, 0.2 mM of dNTPs, 1.5 to 3.5 mM of $MgCl_2$, and 1.25 units of Expand High Fidelity enzyme mix in 1x Expand High Fidelity buffer (Roche Applied Science, Basel, Switzerland). The reactions were performed on a T3 Thermocycler PCR system (Biometra, Göttingen, Germany) using the following conditions: 40 cycles of 94°C for 20 s, 53 to 55°C for 30 s, and 72°C for 45 s and a final extension at 72°C for 10 min. The PCR products were cloned and sequenced.

Phylogenetic analysis

Alignment of the sequences was performed using the MUltiple Sequence Comparison by Log-Expectation program (MUSCLE) (Edgar, 2004). Minor manual adjustments of alignments, trimming, and management of multi-aligned data were carried out using BioEdit v7.0.9 (Hall, 1999). The alignments were illustrated using GeneDoc 2.6 (Nicholas et al., 1997). The Needleman-Wunsch global alignment algorithm (Needleman and Wunsch, 1970) from the

European Molecular Biology Open Software Suite (EMBOSS) was used for the comparison of two sequences (Rice et al., 2000). Unrooted consensus trees were calculated using the PHYLogeny Inference Package (PHYLIP) (Felsenstein, 1989). For each consensus tree, 10,000 bootstrap resamplings of multi-aligned sequences were generated with Seqboot; distance matrices were computed with Dnadist; trees were constructed using the neighbor-joining method (Saitou and Nei, 1987) as implemented in Neighbor; and finally, a consensus tree was built using Consense. Phylogenetic trees were drawn with TreeView (Page, 1996).

GenBank accession numbers

The novel sequences of *Volvox rousseletii* strain MI01 that are described in this study have been deposited under the following accession numbers: *psa*A [GenBank: GU325792], *psa*B [GenBank: GU325794], *rbc*L [GenBank: GU325796], and ITS2 [GenBank: GU325798]. The accession numbers of other cited sequences are given in Additional File 3.

References

- Coleman AW: **Phylogenetic analysis of "Volvocaceae" for comparative genetic studies**. *Proc Natl Acad Sci USA* 1999, **96**:13892-13897.

- Coleman AW, Mai JC: **Ribosomal DNA ITS-1 and ITS-2 sequence comparisons as a tool for predicting genetic relatedness**. *J Mol Evol* 1997, **45**:168-177.

- Coleman AW, Preparata RM, Mehrotra B, Mai JC: **Derivation of the secondary structure of the ITS-1 transcript in Volvocales and its taxonomic correlations**. *Protist* 1998, **149**:135-146.

- Coleman AW, Suarez A, Goff LJ: **Molecular delineation of species and syngens in volvocacean green algae (Chlorophyta)**. *J Phycol* 1994, **30**:80-90.

- Edgar RC: **MUSCLE: multiple sequence alignment with high accuracy and high throughput**. *Nucleic Acids Res* 2004, **32**:1792-1797.

- Felsenstein J: **Phylip - Phylogeny Inference Package (Version 3.2)**. *Cladistics* 1989, **5**:164-166.

- Hall TA: **BioEdit: a user-friendly biological sequence alignment editor and analysis program for Windows 95/98/NT**. *Nucleic Acids Symp Ser* 1999, **41**:95-98.

- Kirk DL: ***Volvox*: molecular-genetic origins of multicellularity and cellular differentiation**. Cambridge: Cambridge University Press; 1998.

- Mai JC, Coleman AW: **The internal transcribed spacer 2 exhibits a common secondary structure in green algae and flowering plants**. *J Mol Evol* 1997, **44**:258-271.

- Needleman SB, Wunsch CD: **A general method applicable to the search for similarities in the amino acid sequence of two proteins**. *J Mol Biol* 1970, **48**:443-453.

- Nicholas KB, Nicholas HB, Deerfield DW: **GeneDoc: Analysis and visualization of genetic variation**. *EMBnetnews* 1997, **4**:14.

- Nozaki H: **Origin and evolution of the genera *Pleodorina* and *Volvox* (Volvocales)**. *Biologia (Bratisl)* 2003, **58**:425-431.

- Nozaki H, Misawa K, Kajita T, Kato M, Nohara S, Watanabe MM: **Origin and evolution of the colonial Volvocales (Chlorophyceae) as inferred from multiple, chloroplast gene sequences**. *Mol Phylogenet Evol* 2000, **17**:256-268.

- Page RD: **TreeView: an application to display phylogenetic trees on personal computers**. *Comput Appl Biosci* 1996, **12**:357-358.

- Rice P, Longden I, Bleasby A: **EMBOSS: the European Molecular Biology Open Software Suite**. *Trends Genet* 2000, **16**:276-277.

- Saitou N, Nei M: **The neighbor-joining method: a new method for reconstructing phylogenetic trees**. *Mol Biol Evol* 1987, **4**:406-425.
