# Supplementary material for: How 5000 independent rowers coordinate their strokes in order to row into the sunlight: Phototaxis in the multicellular green alga Volvox
Source: BMC Biol. 2010 Jul 27;8:103. doi: 10.1186/1741-7007-8-103 (PMC2920248; doi:10.1186/1741-7007-8-103)
Supplement: Additional file 3 — List of species, strains, abbreviations, accession numbers, and references for the psaA, psaB, rbcL and ITS2 sequences from volvocine species. [file 1741-7007-8-103-S3.PDF]

List of species, strains, abbreviations, accession numbers, and references for the *psa*A, *psa*B, *rbc*L and ITS2 sequences from volvocine species

*psa*A, *psa*B, *rbc*L sequences

Species	Strain	Abbreviation	Accession numbers			References
			*psa*A	*psa*B	*rbc*L	
**Volvocaceae**						
Volvox rousseletii	MI01	*Vorou*01	GU325792	GU325794	GU325796	a)
Volvox rousseletii section	UTEX 1862	*Vorou*1862	AB044188	AB044429	D63448	b)
Volvox barberi *Volvox*	UTEX 804	*Vobar*	AB044186	AB044427	D86835	b)
Volvox globator	SAG 199.80	*Voglo*80	GU325793	GU325795	GU325797	a)
Volvox globator	UTEX 955	*Voglo*955	AB044187	AB044428	D86836	b)
Volvox aureus	NIES-541	*Voaur*541	AB044182	AB044424	D63445	b)
Volvox aureus	NIES-1156	*Voaur*1156	AB076123	AB076145	AB076096	c)
Volvox aureus	NIES-1157	*Voaur*1157	AB076124	AB076146	AB076086	c)
Volvox tertius	UTEX 132	*Voter*	AB076126	AB076147	AB076098	c)
Volvox carteri	NIES-732	*Vocar*732	AB044185	AB044425	D63446	b)
Volvox carteri	UTEX 1885	*Vocar*1885	AB076128	AB076148	AB076099	c)
Volvox carteri	UTEX 1875	*Vocar*1875	AB076130	AB076149	AB076100	c)
Volvox gigas	UTEX 1895	*Vogig*	AB076132	AB076150	AB076084	c)
Volvox obversus	UTEX 1865	*Voobv*	AB076133-<br>AB076136	AB076151	AB076085	c)
Volvox africanus	UTEX 1891	*Voafr*	AB076137	AB076152	AB076101	c)
Volvox dissipatrix	UTEX 2184	*Vodis*	AB044183	AB044426	D63447	b)
Pleodorina californica	UTEX 809	*Plcal*	AB044192	AB044430	D63439	b)
Pleodorina japonica	UTEX 2523	*Pljap*	AB044194	AB044431	D63440	b)
Pleodorina indica	UTEX 1990	*Plind*1990	AB044197	AB044432,<br>AB044433	D86834	b)
Eudorina cylindrica	UTEX 1197	*Eucyl*1197	AB044210	AB044441	D86833	b)
Eudorina unicocca	UTEX 1215	*Euuni*	AB044209	AB044440	D63434	b)
Eudorina elegans	NIES-456	*Euele*456	AB044199	AB044435	D63432	b)
Eudorina illinoisensis	NIES-460	*Euill*460	AB044198	AB044434	D63433	b)
Pandorina morum	NIES-574	*Pamor*574	AB044226	AB044452	D63442	b)
Pandorina colemaniae	NIES-572	*Pacol*572	AB044232	AB044457	D63441	b)
Volvulina pringsheimii	UTEX 1020	*Vupri*	AB044220	AB044447	D63444	b)
Volvulina compacta	NIES-582	*Vucom*582	AB044219	AB044446	D86832	b)
Volvulina steinii	UTEX 1525	*Vuste*	AB044223	AB044449	AB044160	b)
Volvulina boldii	UTEX 2185	*Vubol*2185	AB044225	AB044451	AB044163	b)
Yamagishiella unicocca	UTEX 2428	*Yauni*2428	AB044213	AB044443	D86823	b)
Platydorina caudata	UTEX 1658	*Ptcau*	AB044212	AB044442	D86828	b)
**Tetrabaenaceae**						
Tetrabaena socialis	NIES-571	*Tesoc*	AB044415	AB044466	D63443	b)
Basichlamys sacculifera	NIES-566	*Basac*566	AB044416	AB044467,<br>AB044468	D63430	b)
**Goniaceae**						
Gonium pectorale	NIES-569	*Gopec*569	AB044242	AB044463	D63437	b)
Gonium octonarium	GO-LC-1+	*Gooct*	AB044241	AB044462	D63436	b)
Gonium quadratum	NIES-653	*Goqua*653	AB044243	AB044464	D63438	b)
Gonium multicoccum	UTEX 2580	*Gomul*2580	AB044240	AB044461	D63435	b)
Gonium viridistellatum	UTEX 2519	*Govir*2519	AB044244	AB044465	D86831	b)
Astrephomene gubernaculifera	UTEX 1394	*Asgub*1394	AB044235	AB044459	AB044170	b)
Astrephomene perforata	NIES-564	*Asper*564	AB044238	AB044460	D63429	b)
**Chlamydomonadaceae**						
Chlamydomonas reinhardtii	137C	*Chrei*137c	AB044419	AB044470	J01399	b)
Chlamydomonas debaryana	UTEX 1344	*Chdeb*1344	AB044417,<br>AB044418	AB044469	D86838	b)
Vitreochlamys ordinata	Nozaki S-4	*Viord*	AB044420	AB044471	AB014041	b)
Vitreochlamys pinguis	NIES-1148	*Vipin*	AB076142	AB076157	AB050491	c)
Vitreochlamys aulata	SAG 69.72	*Viaul*	AB076144	AB076159	AB050492	c)
Lobomonas monstruosa	NIES-474	*Lomon*	AB044421	AB044472	AB044171	b)
**Tetrasporaceae**						
Paulschulzia pseudovolvox	UTEX 167	*Pupse*	AB044422,<br>AB044423	AB044473	D86837	b)

ITS2 sequences

Species		Strain	Accession numbers	References
			ITS2	
**Volvocaceae**				
Volvox rousseletii		MI01	GU325798	a)
Volvox rousseletii		UTEX 1862	U67025	d)
Volvox barberi	section	UTEX 804	U67013	d)
Volvox capensis	*Volvox*	zyg-6	U67014	d)
Volvox globator		SAG 199.80	GU325799	a)
Volvox globator		UTEX 955	U67022	d)
Volvox aureus		UTEX 1899	U67012	d)
Volvox tertius		UTEX 132	U67029	d)
Volvox carteri		Kawa (Starr)	U67018	d)
Volvox carteri		UTEX 1874	U67015	d)
Volvox carteri		UTEX 1876	U67016	d)
Volvox gigas		UTEX 1895	U67021	d)
Volvox obversus		UTEX 1865	U67023	d)
Volvox africanus		UTEX 1891	U67010	d)
Volvox dissipatrix		UTEX 2184	AF182442	e)
Pleodorina californica		UTEX 809	U67003	d)
Pleodorina japonica		UTEX 2523	U67005	d)
Pleodorina indica		ASW 05153	AF098176	f)
Eudorina cylindrica		ASW 05147	AF098171	f)
Eudorina unicocca		UTEX 1215	AF486525	g)
Eudorina elegans		UTEX 1201	U66958	d)
Eudorina illinoisensis		ASW 05144	AF098174	f)
Pandorina morum		UTEX 1732	U66987	d)
Pandorina colemaniae		Japan	AY033910	h)
Volvulina pringsheimii		UTEX 1020	U67032	d)
Volvulina compacta		NIES-583	AF375590	h)
Volvulina steinii		UTEX 1525	U67033	d)
Volvulina boldii		UTEX 2186	AF375589	h)
Yamagishiella unicocca		UTEX 165	AF375783	h)
Platydorina caudata		UTEX 1658	U67000	d)
**Tetrabaenaceae**				
Tetrabaena socialis		NIES-571	U66977	d)
Basichlamys sacculifera		UTEX 822	U66972	d)
**Goniaceae**				
Gonium pectorale		UTEX 826	U23534	i)
Gonium octonarium		LC-1	AF054423	j)
Gonium quadratum		Cat	AF182431	e)
Gonium multicoccum		UTEX 783	U66967	d)
Gonium viridistellatum		UTEX 2520	AF182432	e)
Astrephomene gubernaculifera		UTEX 1393	AF054422	j)
Astrephomene perforata		UTEX 2475	U66939	d)
**Chlamydomonadaceae**				
Chlamydomonas reinhardtii		CC620	U66954	d)
Chlamydomonas debaryana		CCAP 11/130	AJ749627	k)
Vitreochlamys ordinata		Nozaki S-4	AJ749611	k)

References

a) This study.

b) Nozaki H, Misawa K, Kajita T, Kato M, Nohara S, Watanabe MM: **Origin and evolution of the colonial Volvocales (Chlorophyceae) as inferred from multiple, chloroplast gene sequences**. *Mol Phylogenet Evol* 2000, **17**:256-268.

c) Nozaki H, Takahara M, Nakazawa A, Kita Y, Yamada T, Takano H, Kawano S, Kato M: **Evolution of rbcL group IA introns and intron open reading frames within the colonial Volvocales (Chlorophyceae)**. *Mol Phylogenet Evol* 2002, **23**:326-338.

d) Mai JC, Coleman AW: **The internal transcribed spacer 2 exhibits a common secondary structure in green algae and flowering plants**. *J Mol Evol* 1997, **44**:258-271.

e) Coleman AW: **Phylogenetic analysis of "Volvocaceae" for comparative genetic studies**. *Proc Natl Acad Sci USA* 1999, **96**:13892-13897.

f) Coleman AW: 1998.

g) Coleman AW: **Comparison of *Eudorina* / *Pleodorina* ITS sequences of isolates from nature with those from experimental hybrids**. *Am J Bot* 2002, **89**:1523-1530.

h) Coleman AW: **Biogeography and speciation in the *Pandorina / Volvulina* superclade**. *J Phycol* 2001, **37**:836-851.

i) Coleman AW, Suarez A, Goff LJ: **Molecular delineation of species and syngens in volvocacean green algae (Chlorophyta)**. *J Phycol* 1994, **30**:80-90.

j) Coleman AW, Preparata RM, Mehrotra B, Mai JC: **Derivation of the secondary structure of the ITS-1 transcript in Volvocales and its taxonomic correlations**. *Protist* 1998, **149**:135-146.

k) Pröschold T, Harris EH, Coleman AW: 2006.
