# Supplementary material for: How 5000 independent rowers coordinate their strokes in order to row into the sunlight: Phototaxis in the multicellular green alga Volvox
Source: BMC Biol. 2010 Jul 27;8:103. doi: 10.1186/1741-7007-8-103 (PMC2920248; doi:10.1186/1741-7007-8-103)
Supplement: Additional file 4 — Sequence alignment of psaA cDNA fragments from several volvocine species. [file 1741-7007-8-103-S4.PDF]

Sequence alignment of *psa*A cDNA fragments from several volvocine species

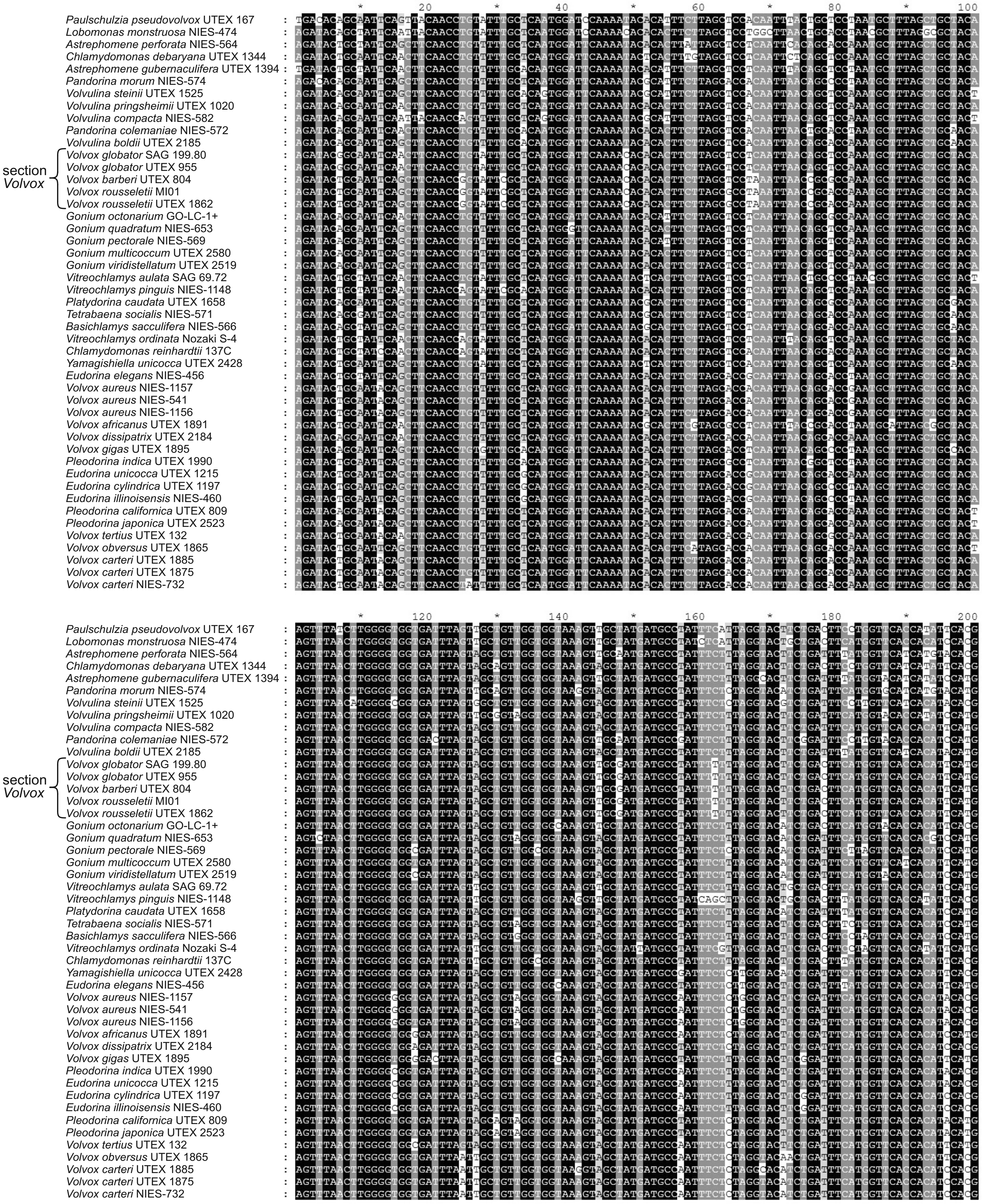

Sequence alignment of *psa*A cDNA fragments from several volvocine species

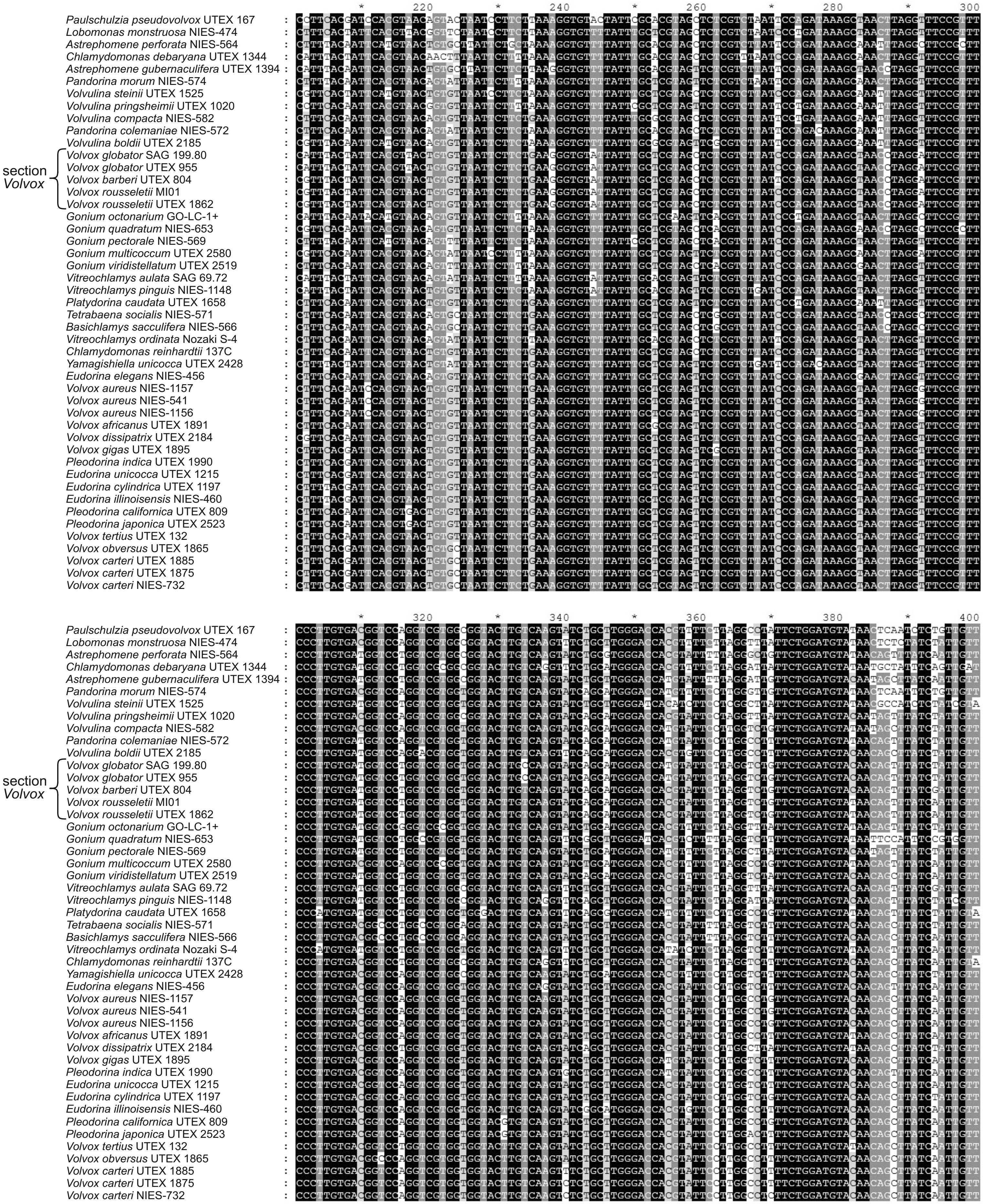

Sequence alignment of *psa*A cDNA fragments from several volvocine species

			*
	Paulschulzia pseudovolvox UTEX 167	:	ATTTTCCACTTTAGC
	Lobomonas monstruosa NIES-474	:	ATTTTCCACTTCAGC
	Astrephomene perforata NIES-564	:	ATTTTCCACTTTAGT
	Chlamydomonas debaryana UTEX 1344	:	ATTTTCCACTTTAGT
	Astrephomene gubernaculifera UTEX 1394	:	ATTTTCCACTTCAGC
	Pandorina morum NIES-574	:	ATTTTCCACTTTAGT
	Volvulina steinii UTEX 1525	:	ATTTTCCACTTTAGT
	Volvulina pringsheimii UTEX 1020	:	ATTTTCCATTTTAGC
	Volvulina compacta NIES-582	:	ATTTTCCACTTTAGT
	Pandorina colemaniae NIES-572	:	ATTTTCCACTTTAGC
	Volvulina boldii UTEX 2185	:	ATTTTCCACTTTAGC
section *Volvox*	*Volvox globator* SAG 199.80	:	ATTTTCCACTTTAGT
section *Volvox*	*Volvox globator* UTEX 955	:	ATTTTCCACTTTAGT
section *Volvox*	*Volvox barberi* UTEX 804	:	ATTTTCCACTTTAGC
section *Volvox*	*Volvox rousseletii* MI01	:	ATTTTCCACTTTAGC
section *Volvox*	*Volvox rousseletii* UTEX 1862	:	ATTTTCCACTTTAGC
	Gonium octonarium GO-LC-1+	:	ATTTTCCACTTCAGC
	Gonium quadratum NIES-653	:	ATTTTCCACTTTAGC
	Gonium pectorale NIES-569	:	ATTTTCCACTTCAGT
	Gonium multicoccum UTEX 2580	:	ATTTTCCACTTCAGC
	Gonium viridistellatum UTEX 2519	:	ATTTTCCACTTCAGC
	Vitreochlamys aulata SAG 69.72	:	ATTTTCCACTTTAGC
	Vitreochlamys pinguis NIES-1148	:	ATTTTCCACTTCAGC
	Platydorina caudata UTEX 1658	:	ATTTTCCACTTTAGT
	Tetrabaena socialis NIES-571	:	ATTTTCCACTTCAGC
	Basichlamys sacculifera NIES-566	:	ATTTTCCACTTTAGT
	Vitreochlamys ordinata Nozaki S-4	:	ATTTTCCACTTTAGT
	Chlamydomonas reinhardtii 137C	:	ATTTTCCACTTCAGC
	Yamagishiella unicocca UTEX 2428	:	ATTTTCCACTTCAGC
	Eudorina elegans NIES-456	:	ATTTTCCACTTCAGC
	Volvox aureus NIES-1157	:	ATTTTCCACTTTAGC
	Volvox aureus NIES-541	:	ATTTTCCACTTTAGC
	Volvox aureus NIES-1156	:	ATTTTCCACTTTAGC
	Volvox africanus UTEX 1891	:	ATTTTCCACTTCAGC
	Volvox dissipatrix UTEX 2184	:	ATTTTCCACTTCAGT
	Volvox gigas UTEX 1895	:	ATTTTCCACTTTAGC
	Pleodorina indica UTEX 1990	:	ATTTTCCACTTTAGC
	Eudorina unicocca UTEX 1215	:	ATTTTCCACTTCAGC
	Eudorina cylindrica UTEX 1197	:	ATTTTCCACTTTAGC
	Eudorina illinoisensis NIES-460	:	ATTTTCCACTTCAGC
	Pleodorina californica UTEX 809	:	ATTTTCCACTTCAGC
	Pleodorina japonica UTEX 2523	:	ATTTTCCACTTCAGC
	Volvox tertius UTEX 132	:	ATTTTCCACTTTAGC
	Volvox obversus UTEX 1865	:	ATTTTCCACTTCAGC
	Volvox carteri UTEX 1885	:	ATTTTCCACTTCAGC
	Volvox carteri UTEX 1875	:	ATTTTCCACTTCAGC
	Volvox carteri NIES-732	:	ATTTTCCACTTCAGC

Alignment of sequences was done using the MUltiple Sequence Comparison by Log-Expectation program (MUSCLE) (Edgar, 2004). Conserved nucleotides were shaded using GeneDoc 2.6 (Nicholas et al., 1997). White letters on black background: conserved in 100 percent of the sequences at the corresponding position; white letters on dark gray background: conserved in >80 percent of the sequences at the corresponding position; black letters on light gray background: conserved in >60 percent of the sequences at the corresponding position.

References

- Edgar RC: **MUSCLE: multiple sequence alignment with high accuracy and high throughput**. *Nucleic Acids Res* 2004, **32**:1792-1797.

- Nicholas KB, Nicholas HB, Deerfield DW: **GeneDoc: Analysis and visualization of genetic variation**. *EMBnetnews* 1997, **4**:14.
