# Supplementary material for: How 5000 independent rowers coordinate their strokes in order to row into the sunlight: Phototaxis in the multicellular green alga Volvox
Source: BMC Biol. 2010 Jul 27;8:103. doi: 10.1186/1741-7007-8-103 (PMC2920248; doi:10.1186/1741-7007-8-103)
Supplement: Additional file 6 — Sequence alignment of rbcL cDNA fragments from several volvocine species. [file 1741-7007-8-103-S6.PDF]

Sequence alignment of *rbc*L cDNA fragments from several volvocine species

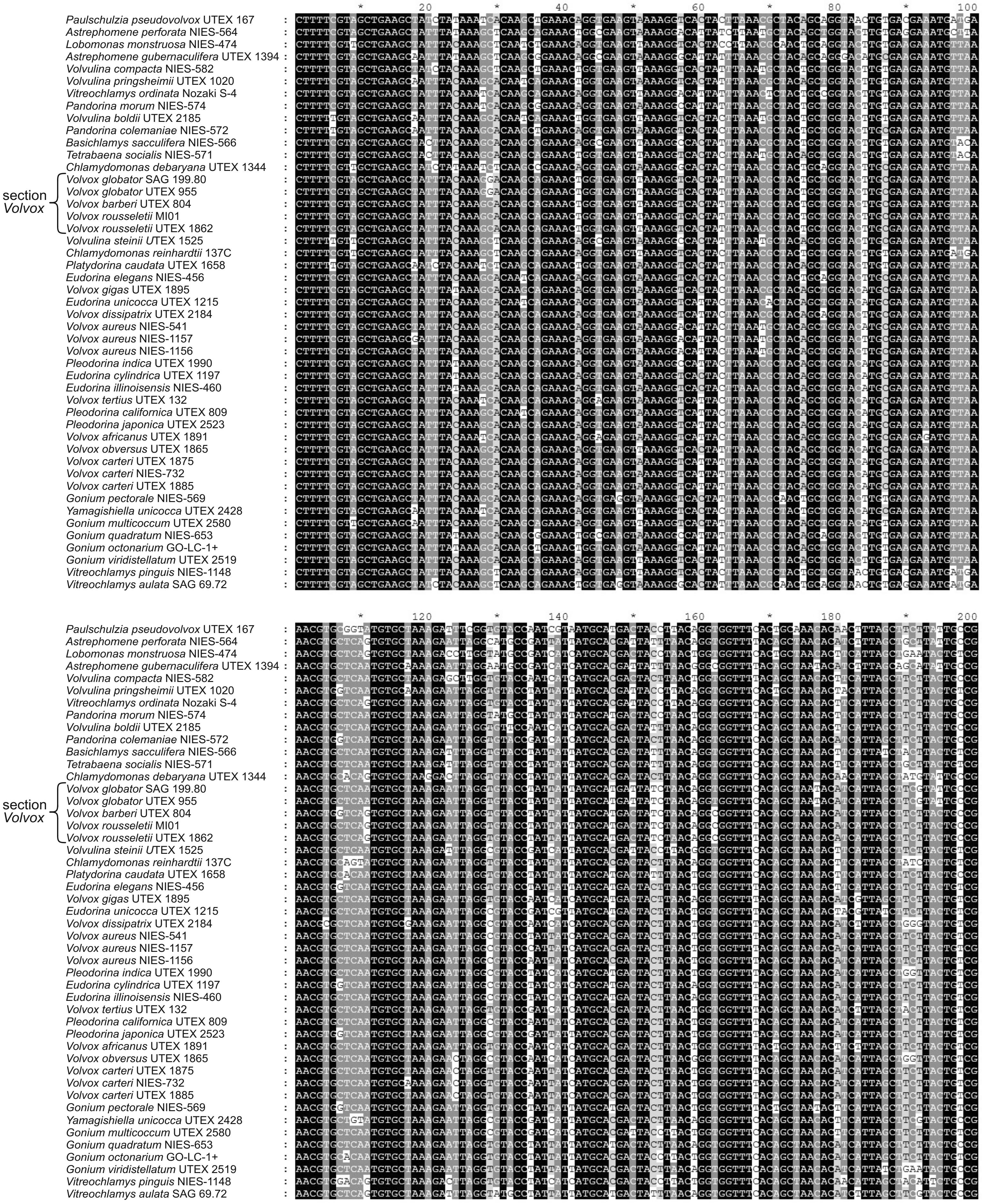

Sequence alignment of *rbc*L cDNA fragments from several volvocine species

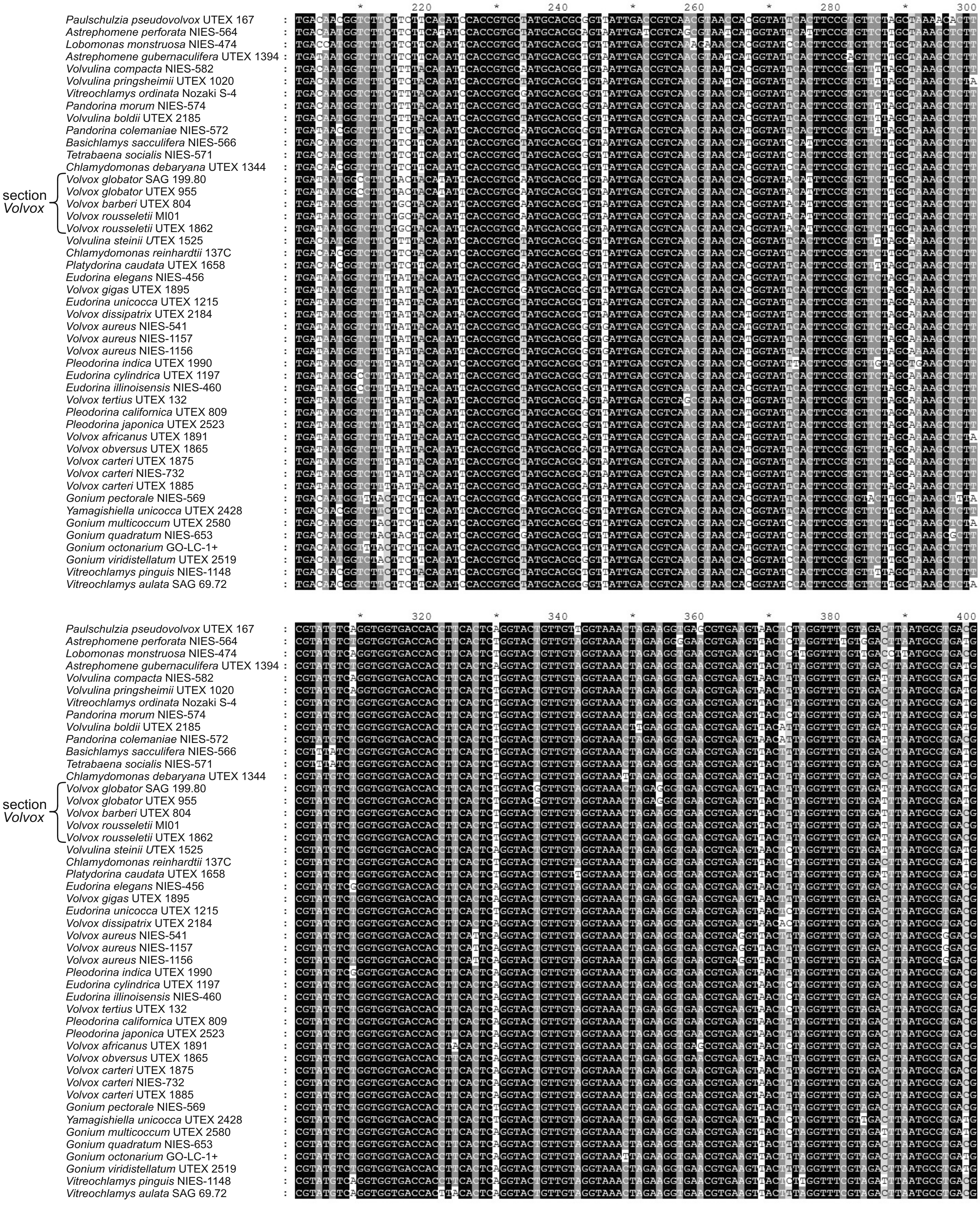

Sequence alignment of *rbc*L cDNA fragments from several volvocine species

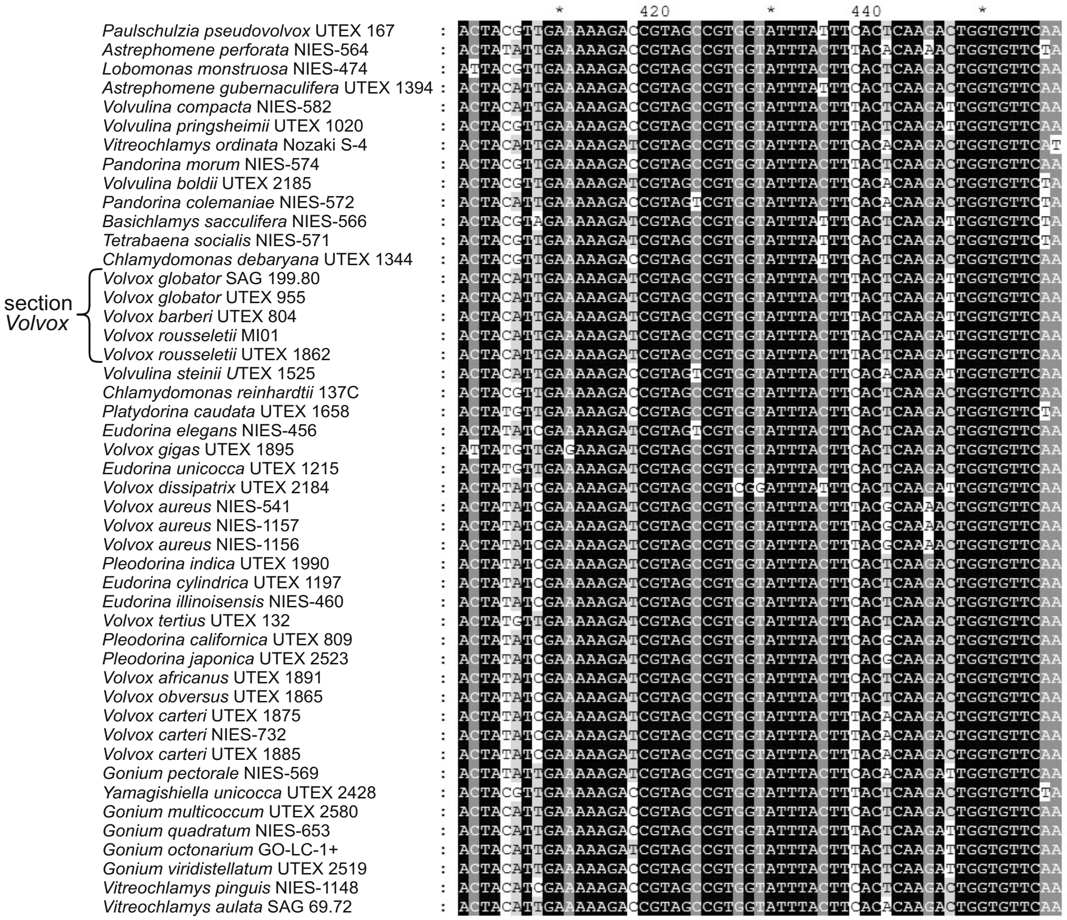

Alignment of sequences was done using the MUltiple Sequence Comparison by Log-Expectation program (MUSCLE) (Edgar, 2004). Conserved nucleotides were shaded using GeneDoc 2.6 (Nicholas et al., 1997). White letters on black background: conserved in 100 percent of the sequences at the corresponding position; white letters on dark gray background: conserved in >80 percent of the sequences at the corresponding position; black letters on light gray background: conserved in >60 percent of the sequences at the corresponding position.

References

- Edgar RC: **MUSCLE: multiple sequence alignment with high accuracy and high throughput**. *Nucleic Acids Res* 2004, **32**:1792-1797.

- Nicholas KB, Nicholas HB, Deerfield DW: **GeneDoc: Analysis and visualization of genetic variation**. *EMBnetnews* 1997, **4**:14.
