# Supplementary material for: How 5000 independent rowers coordinate their strokes in order to row into the sunlight: Phototaxis in the multicellular green alga Volvox
Source: BMC Biol. 2010 Jul 27;8:103. doi: 10.1186/1741-7007-8-103 (PMC2920248; doi:10.1186/1741-7007-8-103)
Supplement: Additional file 7 — Comparison of psaA sequences from several volvocine species. [file 1741-7007-8-103-S7.PDF]

Comparison of *psa*A sequences from several volvocine species

				***psa*A**		
**Compared species**				**Identities**	**Gaps**	**Expect**
Volvox rousseletii MI01	vs.	*Volvox rousseletii* MI01		415/415 (100.00%)	0/415 (0.00%)	0
Volvox rousseletii MI01	vs.	*Volvox rousseletii* UTEX 1862		415/415 (100.00%)	0/415 (0.00%)	0
Volvox rousseletii MI01	vs.	*Volvox barberi* UTEX 804	section *Volvox*	410/415 (98.80%)	0/415 (0.00%)	0
Volvox rousseletii MI01	vs.	*Volvox globator* SAG 199.80		400/414 (96.62%)	0/414 (0.00%)	0
Volvox rousseletii MI01	vs.	*Volvox globator* UTEX 955		400/414 (96.62%)	0/414 (0.00%)	0
Volvox rousseletii MI01	vs.	*Volvox tertius* UTEX-132		381/415 (91.81%)	0/415 (0.00%)	2.00E-174
Volvox rousseletii MI01	vs.	*Gonium viridistellatum* UTEX 2519		380/415 (91.57%)	0/415 (0.00%)	7.00E-173
Volvox rousseletii MI01	vs.	*Chlamydomonas reinhardtii* 137C		380/415 (91.57%)	0/415 (0.00%)	7.00E-173
Volvox rousseletii MI01	vs.	*Volvox dissipatrix* UTEX 2184		379/414 (91.55%)	0/414 (0.00%)	3.00E-172
Volvox rousseletii MI01	vs.	*Eudorina cylindrica* UTEX 1197		379/415 (91.33%)	0/415 (0.00%)	9.00E-172
Volvox rousseletii MI01	vs.	*Vitreochlamys aulata* SAG 69.72		379/415 (91.33%)	0/415 (0.00%)	9.00E-172
Volvox rousseletii MI01	vs.	*Basichlamys sacculifera* NIES-566		377/414 (91.06%)	0/414 (0.00%)	1.00E-169
Volvox rousseletii MI01	vs.	*Eudorina elegans* NIES-456		377/415 (90.84%)	0/415 (0.00%)	5.00E-169
Volvox rousseletii MI01	vs.	*Eudorina illinoisensis* NIES-460		377/415 (90.84%)	0/415 (0.00%)	5.00E-169
Volvox rousseletii MI01	vs.	*Tetrabaena socialis* NIES-571		376/415 (90.60%)	0/415 (0.00%)	2.00E-167
Volvox rousseletii MI01	vs.	*Pleodorina californica* UTEX 809		376/415 (90.60%)	0/415 (0.00%)	2.00E-167
Volvox rousseletii MI01	vs.	*Volvox aureus* NIES-541		376/415 (90.60%)	0/415 (0.00%)	2.00E-167
Volvox rousseletii MI01	vs.	*Volvox aureus* NIES-1156		376/415 (90.60%)	0/415 (0.00%)	2.00E-167
Volvox rousseletii MI01	vs.	*Volvox aureus* NIES-1157		376/415 (90.60%)	0/415 (0.00%)	2.00E-167
Volvox rousseletii MI01	vs.	*Volvox gigas* UTEX1895		376/415 (90.60%)	0/415 (0.00%)	2.00E-167
Volvox rousseletii MI01	vs.	*Volvox africanus* UTEX1891		376/415 (90.60%)	0/415 (0.00%)	2.00E-167
Volvox rousseletii MI01	vs.	*Vitreochlamys pinguis* NIES-1148		376/415 (90.60%)	0/415 (0.00%)	2.00E-167
Volvox rousseletii MI01	vs.	*Vitreochlamys ordinata* Nozaki S-4		375/414 (90.58%)	0/414 (0.00%)	7.00E-167
Volvox rousseletii MI01	vs.	*Gonium octonarium* GO-LC-1+		375/415 (90.36%)	0/415 (0.00%)	2.00E-166
Volvox rousseletii MI01	vs.	*Yamagishiella unicocca* UTEX 2428		374/415 (90.12%)	0/415 (0.00%)	1.00E-164
Volvox rousseletii MI01	vs.	*Volvox obversus* UTEX1865		373/415 (89.88%)	0/415 (0.00%)	1.00E-163
Volvox rousseletii MI01	vs.	*Volvulina boldii* UTEX 2185		372/415 (89.64%)	0/415 (0.00%)	5.00E-162
Volvox rousseletii MI01	vs.	*Gonium pectorale* NIES-569		371/414 (89.61%)	0/414 (0.00%)	2.00E-161
Volvox rousseletii MI01	vs.	*Astrephomene gubernaculifera* UTEX 1394		370/414 (89.37%)	0/414 (0.00%)	2.00E-160
Volvox rousseletii MI01	vs.	*Pandorina colemaniae* NIES-572		370/415 (89.16%)	0/415 (0.00%)	3.00E-159
Volvox rousseletii MI01	vs.	*Gonium quadratum* NIES-653		369/415 (88.92%)	0/415 (0.00%)	3.00E-158
Volvox rousseletii MI01	vs.	*Volvulina pringsheimii* UTEX 1020		368/415 (88.67%)	0/415 (0.00%)	1.00E-156
Volvox rousseletii MI01	vs.	*Platydorina caudata* UTEX 1658		341/379 (89.97%)	0/379 (0.00%)	2.00E-149
Volvox rousseletii MI01	vs.	*Volvox carteri* UTEX1875		341/379 (89.97%)	0/379 (0.00%)	2.00E-149
Volvox rousseletii MI01	vs.	*Lobomonas monstruosa* NIES-474		362/415 (87.23%)	0/415 (0.00%)	2.00E-148
Volvox rousseletii MI01	vs.	*Chlamydomonas debaryana* UTEX 1344		361/414 (87.20%)	0/414 (0.00%)	7.00E-148
Volvox rousseletii MI01	vs.	*Astrephomene perforata* NIES-564		339/379 (89.45%)	0/379 (0.00%)	1.00E-146
Volvox rousseletii MI01	vs.	*Paulschulzia pseudovolvox* UTEX 167		358/414 (86.47%)	0/414 (0.00%)	4.00E-144
Volvox rousseletii MI01	vs.	*Pandorina morum* NIES-574		357/414 (86.23%)	0/414 (0.00%)	2.00E-142
Volvox rousseletii MI01	vs.	*Volvulina steinii* UTEX 1525		357/414 (86.23%)	0/414 (0.00%)	2.00E-142
Volvox rousseletii MI01	vs.	*Gonium multicoccum* UTEX 2580		208/228 (91.23%)	0/228 (0.00%)	6.00E-92
Volvox rousseletii MI01	vs.	*Eudorina unicocca* UTEX 1215		208/228 (91.23%)	0/228 (0.00%)	6.00E-92
Volvox rousseletii MI01	vs.	*Pleodorina indica* UTEX 1990		207/228 (90.79%)	0/228 (0.00%)	3.00E-90
Volvox rousseletii MI01	vs.	*Volvulina compacta* NIES-582		206/227 (90.75%)	0/227 (0.00%)	1.00E-89
Volvox rousseletii MI01	vs.	*Pleodorina japonica* UTEX 2523		206/228 (90.35%)	0/228 (0.00%)	3.00E-89
Volvox rousseletii MI01	vs.	*Volvox carteri* NIES-732		205/228 (89.91%)	0/228 (0.00%)	1.00E-87
Volvox rousseletii MI01	vs.	*Volvox carteri* UTEX1885		205/228 (89.91%)	0/228 (0.00%)	1.00E-87

Comparison of photosystem I P700 chlorophyll a apoprotein A1 (*psa*A) sequences. The list is sorted by expected value (Expect) in ascending order.
