# Supplementary material for: How 5000 independent rowers coordinate their strokes in order to row into the sunlight: Phototaxis in the multicellular green alga Volvox
Source: BMC Biol. 2010 Jul 27;8:103. doi: 10.1186/1741-7007-8-103 (PMC2920248; doi:10.1186/1741-7007-8-103)
Supplement: Additional file 8 — Comparison of psaB sequences from several volvocine species. [file 1741-7007-8-103-S8.PDF]

Comparison of *psa*B sequences from several volvocine species

Compared species				***psa*B**		
				**Identities**	**Gaps**	**Expect**
Volvox rousseletii MI01	vs.	*Volvox rousseletii* MI01	section *Volvox*	316/316 (100.00%)	0/316 (0.00%)	1.00E-167
Volvox rousseletii MI01	vs.	*Volvox rousseletii* UTEX 1862	section *Volvox*	316/316 (100.00%)	0/316 (0.00%)	5.00E-167
Volvox rousseletii MI01	vs.	*Volvox barberi* UTEX 804	section *Volvox*	310/315 (98.41%)	0/315 (0.00%)	2.00E-159
Volvox rousseletii MI01	vs.	*Volvox globator* SAG 199.80	section *Volvox*	299/316 (94.62%)	0/316 (0.00%)	2.00E-144
Volvox rousseletii MI01	vs.	*Volvox globator* UTEX 955	section *Volvox*	299/316 (94.62%)	0/316 (0.00%)	1.00E-143
Volvox rousseletii MI01	vs.	*Gonium viridistellatum* UTEX 2519		285/315 (90.48%)	0/315 (0.00%)	9.00E-126
Volvox rousseletii MI01	vs.	*Pleodorina indica* UTEX 1990		283/315 (89.84%)	0/315 (0.00%)	5.00E-123
Volvox rousseletii MI01	vs.	*Yamagishiella unicocca* UTEX 2428		282/314 (89.81%)	0/314 (0.00%)	2.00E-122
Volvox rousseletii MI01	vs.	*Volvulina pringsheimii* UTEX 1020		283/316 (89.56%)	0/316 (0.00%)	6.00E-122
Volvox rousseletii MI01	vs.	*Volvox obversus* UTEX1865		282/316 (89.24%)	0/316 (0.00%)	7.00E-121
Volvox rousseletii MI01	vs.	*Volvox carteri* UTEX1875		282/316 (89.24%)	0/316 (0.00%)	7.00E-121
Volvox rousseletii MI01	vs.	*Volvox carteri* NIES-732		282/316 (89.24%)	0/316 (0.00%)	7.00E-121
Volvox rousseletii MI01	vs.	*Volvox carteri* UTEX1885		282/316 (89.24%)	0/316 (0.00%)	7.00E-121
Volvox rousseletii MI01	vs.	*Eudorina unicocca* UTEX 1215		281/315 (89.21%)	0/315 (0.00%)	3.00E-120
Volvox rousseletii MI01	vs.	*Eudorina cylindrica* UTEX 1197		281/316 (88.92%)	0/316 (0.00%)	3.00E-119
Volvox rousseletii MI01	vs.	*Eudorina illinoisensis* NIES-460		280/315 (88.89%)	0/315 (0.00%)	1.00E-118
Volvox rousseletii MI01	vs.	*Volvox gigas* UTEX1895		280/316 (88.61%)	0/316 (0.00%)	4.00E-118
Volvox rousseletii MI01	vs.	*Volvox aureus* NIES-541		279/315 (88.57%)	0/315 (0.00%)	1.00E-117
Volvox rousseletii MI01	vs.	*Gonium pectorale* NIES-569		279/315 (88.57%)	0/315 (0.00%)	1.00E-117
Volvox rousseletii MI01	vs.	*Gonium quadratum* NIES-653		279/315 (88.57%)	0/315 (0.00%)	1.00E-117
Volvox rousseletii MI01	vs.	*Platydorina caudata* UTEX 1658		279/315 (88.57%)	0/315 (0.00%)	1.00E-117
Volvox rousseletii MI01	vs.	*Pleodorina californica* UTEX 809		279/316 (88.29%)	0/316 (0.00%)	2.00E-116
Volvox rousseletii MI01	vs.	*Volvox africanus* UTEX1891		279/316 (88.29%)	0/316 (0.00%)	2.00E-116
Volvox rousseletii MI01	vs.	*Volvulina steinii* UTEX 1525		279/316 (88.29%)	0/316 (0.00%)	2.00E-116
Volvox rousseletii MI01	vs.	*Volvox aureus* NIES-1156		278/315 (88.25%)	0/315 (0.00%)	6.00E-116
Volvox rousseletii MI01	vs.	*Volvox aureus* NIES-1157		278/315 (88.25%)	0/315 (0.00%)	6.00E-116
Volvox rousseletii MI01	vs.	*Gonium octonarium* GO-LC-1+		278/315 (88.25%)	0/315 (0.00%)	6.00E-116
Volvox rousseletii MI01	vs.	*Gonium multicoccum* UTEX 2580		278/315 (88.25%)	0/315 (0.00%)	6.00E-116
Volvox rousseletii MI01	vs.	*Volvox tertius* UTEX-132		278/316 (87.97%)	0/316 (0.00%)	2.00E-115
Volvox rousseletii MI01	vs.	*Astrephomene gubernaculifera* UTEX 1394		277/314 (88.22%)	0/314 (0.00%)	2.00E-115
Volvox rousseletii MI01	vs.	*Pleodorina japonica* UTEX 2523		278/316 (87.97%)	0/316 (0.00%)	2.00E-115
Volvox rousseletii MI01	vs.	*Pandorina colemaniae* NIES-572		277/316 (87.66%)	0/316 (0.00%)	8.00E-114
Volvox rousseletii MI01	vs.	*Volvulina boldii* UTEX 2185		276/315 (87.62%)	0/315 (0.00%)	3.00E-113
Volvox rousseletii MI01	vs.	*Eudorina elegans* NIES-456		276/316 (87.34%)	0/316 (0.00%)	1.00E-112
Volvox rousseletii MI01	vs.	*Volvulina compacta* NIES-582		275/314 (87.58%)	0/314 (0.00%)	1.00E-112
Volvox rousseletii MI01	vs.	*Volvox dissipatrix* UTEX 2184		275/315 (87.30%)	0/315 (0.00%)	4.00E-112
Volvox rousseletii MI01	vs.	*Pandorina morum* NIES-574		275/317 (86.75%)	6/317 (1.89%)	2.00E-109
Volvox rousseletii MI01	vs.	*Vitreochlamys ordinata* Nozaki S-4		272/316 (86.08%)	0/316 (0.00%)	3.00E-107
Volvox rousseletii MI01	vs.	*Vitreochlamys pinguis* NIES-1148		271/316 (85.76%)	0/316 (0.00%)	1.00E-105
Volvox rousseletii MI01	vs.	*Astrephomene perforata* NIES-564		270/315 (85.71%)	0/315 (0.00%)	4.00E-105
Volvox rousseletii MI01	vs.	*Chlamydomonas reinhardtii* 137C		270/316 (85.44%)	0/316 (0.00%)	1.00E-104
Volvox rousseletii MI01	vs.	*Lobomonas monstruosa* NIES-474		271/317 (85.49%)	4/317 (1.26%)	2.00E-103
Volvox rousseletii MI01	vs.	*Vitreochlamys aulata* SAG 69.72		269/316 (85.13%)	0/316 (0.00%)	6.00E-103
Volvox rousseletii MI01	vs.	*Basichlamys sacculifera* NIES-566		268/315 (85.08%)	0/315 (0.00%)	2.00E-102
Volvox rousseletii MI01	vs.	*Tetrabaena socialis* NIES-571		268/315 (85.08%)	0/315 (0.00%)	2.00E-102
Volvox rousseletii MI01	vs.	*Chlamydomonas debaryana* UTEX 1344		265/314 (84.39%)	0/314 (0.00%)	4.00E-99
Volvox rousseletii MI01	vs.	*Paulschulzia pseudovolvox* UTEX 167		264/316 (83.54%)	0/316 (0.00%)	2.00E-96

Comparison of photosystem I P700 chlorophyll a apoprotein A2 (*psa*B) sequences. The list is sorted by expected value (Expect) in ascending order.
