# Supplementary material for: How 5000 independent rowers coordinate their strokes in order to row into the sunlight: Phototaxis in the multicellular green alga Volvox
Source: BMC Biol. 2010 Jul 27;8:103. doi: 10.1186/1741-7007-8-103 (PMC2920248; doi:10.1186/1741-7007-8-103)
Supplement: Additional file 9 — Comparison of rbcL sequences from several volvocine species. [file 1741-7007-8-103-S9.PDF]

Comparison of *rbc*L sequences from several volvocine species

Compared species				*rbc*L		
				Identities	Gaps	Expect
Volvox rousseletii MI01	vs.	*Volvox rousseletii* MI01	section *Volvox*	457/457 (100.00%)	0/457 (0.00%)	0
Volvox rousseletii MI01	vs.	*Volvox rousseletii* UTEX 1862	section *Volvox*	457/457 (100.00%)	0/457 (0.00%)	0
Volvox rousseletii MI01	vs.	*Volvox barberi* UTEX 804	section *Volvox*	455/457 (99.56%)	0/457 (0.00%)	0
Volvox rousseletii MI01	vs.	*Volvox globator* SAG 199.80	section *Volvox*	443/457 (96.94%)	0/457 (0.00%)	0
Volvox rousseletii MI01	vs.	*Volvox globator* UTEX 955	section *Volvox*	443/457 (96.94%)	0/457 (0.00%)	0
Volvox rousseletii MI01	vs.	*Gonium viridistellatum* UTEX 2519		430/457 (94.09%)	0/457 (0.00%)	0
Volvox rousseletii MI01	vs.	*Gonium quadratum* NIES-653		429/457 (93.87%)	0/457 (0.00%)	0
Volvox rousseletii MI01	vs.	*Pandorina morum* NIES-574		429/457 (93.87%)	0/457 (0.00%)	0
Volvox rousseletii MI01	vs.	*Vitreochlamys ordinata* Nozaki S-4		427/456 (93.64%)	0/456 (0.00%)	0
Volvox rousseletii MI01	vs.	*Gonium octonarium* GO-LC-1+		427/457 (93.44%)	0/457 (0.00%)	0
Volvox rousseletii MI01	vs.	*Volvulina compacta* NIES-582		427/457 (93.44%)	0/457 (0.00%)	0
Volvox rousseletii MI01	vs.	*Eudorina elegans* NIES-456		426/457 (93.22%)	0/457 (0.00%)	0
Volvox rousseletii MI01	vs.	*Basichlamys sacculifera* NIES-566		424/455 (93.19%)	0/455 (0.00%)	0
Volvox rousseletii MI01	vs.	*Volvox carteri* UTEX1875		425/457 (93.00%)	0/457 (0.00%)	0
Volvox rousseletii MI01	vs.	*Volvox carteri* UTEX1885		425/457 (93.00%)	0/457 (0.00%)	0
Volvox rousseletii MI01	vs.	*Gonium pectorale* NIES-569		425/457 (93.00%)	0/457 (0.00%)	0
Volvox rousseletii MI01	vs.	*Platydorina caudata* UTEX 1658		423/455 (92.97%)	0/455 (0.00%)	0
Volvox rousseletii MI01	vs.	*Volvox carteri* NIES-732		424/457 (92.78%)	0/457 (0.00%)	0
Volvox rousseletii MI01	vs.	*Eudorina illinoisensis* NIES-460		424/457 (92.78%)	0/457 (0.00%)	0
Volvox rousseletii MI01	vs.	*Pandorina colemaniae* NIES-572		422/455 (92.75%)	0/455 (0.00%)	0
Volvox rousseletii MI01	vs.	*Volvox obversus* UTEX1865		423/457 (92.56%)	0/457 (0.00%)	0
Volvox rousseletii MI01	vs.	*Volvox gigas* UTEX1895		423/457 (92.56%)	0/457 (0.00%)	0
Volvox rousseletii MI01	vs.	*Yamagishiella unicocca* UTEX 2428		421/455 (92.53%)	0/455 (0.00%)	0
Volvox rousseletii MI01	vs.	*Tetrabaena socialis* NIES-571		421/455 (92.53%)	0/455 (0.00%)	0
Volvox rousseletii MI01	vs.	*Volvulina pringsheimii* UTEX 1020		422/457 (92.34%)	0/457 (0.00%)	0
Volvox rousseletii MI01	vs.	*Eudorina cylindrica* UTEX 1197		422/457 (92.34%)	0/457 (0.00%)	0
Volvox rousseletii MI01	vs.	*Pleodorina japonica* UTEX 2523		422/457 (92.34%)	0/457 (0.00%)	0
Volvox rousseletii MI01	vs.	*Pleodorina californica* UTEX 809		421/457 (92.12%)	0/457 (0.00%)	0
Volvox rousseletii MI01	vs.	*Eudorina unicocca* UTEX 1215		420/457 (91.90%)	0/457 (0.00%)	0
Volvox rousseletii MI01	vs.	*Gonium multicoccum* UTEX 2580		420/457 (91.90%)	0/457 (0.00%)	0
Volvox rousseletii MI01	vs.	*Chlamydomonas reinhardtii* 137C		420/457 (91.90%)	0/457 (0.00%)	0
Volvox rousseletii MI01	vs.	*Volvox aureus* NIES-541		419/457 (91.68%)	0/457 (0.00%)	0
Volvox rousseletii MI01	vs.	*Volvox aureus* NIES-1156		419/457 (91.68%)	0/457 (0.00%)	0
Volvox rousseletii MI01	vs.	*Volvox aureus* NIES-1157		418/457 (91.47%)	0/457 (0.00%)	0
Volvox rousseletii MI01	vs.	*Volvox tertius* UTEX-132		418/457 (91.47%)	0/457 (0.00%)	0
Volvox rousseletii MI01	vs.	*Pleodorina indica* UTEX 1990		417/457 (91.25%)	0/457 (0.00%)	0
Volvox rousseletii MI01	vs.	*Vitreochlamys aulata* SAG 69.72		416/457 (91.03%)	0/457 (0.00%)	0
Volvox rousseletii MI01	vs.	*Chlamydomonas debaryana* UTEX 1344		416/457 (91.03%)	0/457 (0.00%)	0
Volvox rousseletii MI01	vs.	*Vitreochlamys pinguis* NIES-1148		415/457 (90.81%)	0/457 (0.00%)	0
Volvox rousseletii MI01	vs.	*Volvulina steinii* UTEX 1525		414/457 (90.59%)	0/457 (0.00%)	0
Volvox rousseletii MI01	vs.	*Volvox africanus* UTEX1891		413/457 (90.37%)	0/457 (0.00%)	0
Volvox rousseletii MI01	vs.	*Volvox dissipatrix* UTEX 2184		413/457 (90.37%)	0/457 (0.00%)	0
Volvox rousseletii MI01	vs.	*Astrephomene gubernaculifera* UTEX 1394		411/457 (89.93%)	0/457 (0.00%)	0
Volvox rousseletii MI01	vs.	*Astrephomene perforata* NIES-564		407/455 (89.45%)	0/455 (0.00%)	3.00E-177
Volvox rousseletii MI01	vs.	*Volvulina boldii* UTEX 2185		379/410 (92.44%)	0/410 (0.00%)	1.00E-175
Volvox rousseletii MI01	vs.	*Lobomonas monstruosa* NIES-474		405/457 (88.62%)	0/457 (0.00%)	6.00E-173
Volvox rousseletii MI01	vs.	*Paulschulzia pseudovolvox* UTEX 167		398/457 (87.09%)	0/457 (0.00%)	4.00E-163

Comparison of ribulose bisphosphate carboxylase (*rbc*L) sequences. The list is sorted by expected value (Expect) in ascending order and by identities (%) in descending order.
