# Supplementary material for: How 5000 independent rowers coordinate their strokes in order to row into the sunlight: Phototaxis in the multicellular green alga Volvox
Source: BMC Biol. 2010 Jul 27;8:103. doi: 10.1186/1741-7007-8-103 (PMC2920248; doi:10.1186/1741-7007-8-103)
Supplement: Additional file 10 — Comparison of ITS2 sequences from several volvocine species. [file 1741-7007-8-103-S10.PDF]

Comparison of ITS2 sequences from several volvocine species

Compared species				ITS2		
				**Identities**	**Gaps**	**Expect**
Volvox rousseletii MI01	vs.	*Volvox rousseletii* MI01		124/124 (100.00%)	0/124 (0.00%)	7.00E-66
Volvox rousseletii MI01	vs.	*Volvox rousseletii* UTEX 1862		120/124 (96.77%)	0/124 (0.00%)	1.00E-58
Volvox rousseletii MI01	vs.	*Volvox barberi* UTEX 804	section	104/120 (86.67%)	2/120 (1.67%)	5.00E-39
Volvox rousseletii MI01	vs.	*Volvox capensis* zyg-6	*Volvox*	103/133 (77.44%)	12/133 (9.02%)	5.00E-26
Volvox rousseletii MI01	vs.	*Volvox globator* UTEX 955		85/106 (80.19%)	6/106 (5.66%)	2.00E-24
Volvox rousseletii MI01	vs.	*Volvox globator* SAG 199.80		85/106 (80.19%)	6/106 (5.66%)	7.00E-24
Volvox rousseletii MI01	vs.	*Gonium pectorale* UTEX 826		31/37 (83.78%)	1/37 (2.70%)	4.00E-07
Volvox rousseletii MI01	vs.	*Gonium quadratum* Cat		31/37 (83.78%)	1/37 (2.70%)	4.00E-07
Volvox rousseletii MI01	vs.	*Chlamydomonas reinhardtii* CC620		30/37 (81.08%)	1/37 (2.70%)	2.00E-05
Volvox rousseletii MI01	vs.	*Gonium multicoccum* UTEX 783		31/39 (79.49%)	3/39 (7.69%)	8.00E-05
Volvox rousseletii MI01	vs.	*Pleodorina japonica* UTEX 2523		26/32 (81.25%)	2/32 (6.25%)	1.00E-04
Volvox rousseletii MI01	vs.	*Tetrabaena socialis* NIES-571		25/31 (80.65%)	3/31 (9.68%)	2.00E-04
Volvox rousseletii MI01	vs.	*Astrephomene gubernaculifera* UTEX 1393		21/25 (84.00%)	0/25 (0.00%)	2.00E-04
Volvox rousseletii MI01	vs.	*Volvulina compacta* NIES-583		15/15 (100.00%)	0/15 (0.00%)	2.00E-04
Volvox rousseletii MI01	vs.	*Yamagishiella unicocca* UTEX 165		20/23 (86.96%)	0/23 (0.00%)	2.00E-04
Volvox rousseletii MI01	vs.	*Eudorina illinoisensis* ASW 05144		15/15 (100.00%)	0/15 (0.00%)	2.00E-04
Volvox rousseletii MI01	vs.	*Volvox gigas* UTEX 1895		14/14 (100.00%)	0/14 (0.00%)	3.00E-04
Volvox rousseletii MI01	vs.	*Volvox obversus* UTEX 1865		24/29 (82.76%)	1/29 (3.45%)	3.00E-04
Volvox rousseletii MI01	vs.	*Eudorina cylindrica* ASW 05147		14/14 (100.00%)	0/14 (0.00%)	7.00E-04
Volvox rousseletii MI01	vs.	*Eudorina unicocca* UTEX 1215		16/17 (94.12%)	0/17 (0.00%)	7.00E-04
Volvox rousseletii MI01	vs.	*Pleodorina indica* ASW 05153		14/14 (100.00%)	0/14 (0.00%)	7.00E-04
Volvox rousseletii MI01	vs.	*Basichlamys sacculifera* UTEX 822		15/16 (93.75%)	0/16 (0.00%)	0.002
Volvox rousseletii MI01	vs.	*Gonium octonarium* LC-1		13/13 (100.00%)	0/13 (0.00%)	0.002
Volvox rousseletii MI01	vs.	*Gonium viridistellatum* UTEX 2520		13/13 (100.00%)	0/13 (0.00%)	0.002
Volvox rousseletii MI01	vs.	*Volvulina boldii* UTEX 2186		11/11 (100.00%)	0/11 (0.00%)	0.03
Volvox rousseletii MI01	vs.	*Astrephomene perforata* UTEX 2475			No significant similarity	
Volvox rousseletii MI01	vs.	*Pandorina morum* UTEX 1732			No significant similarity	
Volvox rousseletii MI01	vs.	*Pandorina colemaniae* Japan			No significant similarity	
Volvox rousseletii MI01	vs.	*Volvulina pringsheimii* UTEX 1020			No significant similarity	
Volvox rousseletii MI01	vs.	*Volvulina steinii* UTEX 1525			No significant similarity	
Volvox rousseletii MI01	vs.	*Platydorina caudata* UTEX 1658			No significant similarity	
Volvox rousseletii MI01	vs.	*Eudorina elegans* UTEX 1201			No significant similarity	
Volvox rousseletii MI01	vs.	*Pleodorina californica* UTEX 809			No significant similarity	
Volvox rousseletii MI01	vs.	*Volvox aureus* UTEX 1899			No significant similarity	
Volvox rousseletii MI01	vs.	*Volvox tertius* UTEX 132			No significant similarity	
Volvox rousseletii MI01	vs.	*Volvox carteri* Kawa (Starr)			No significant similarity	
Volvox rousseletii MI01	vs.	*Volvox carteri* UTEX 1874			No significant similarity	
Volvox rousseletii MI01	vs.	*Volvox carteri* UTEX 1876			No significant similarity	
Volvox rousseletii MI01	vs.	*Volvox africanus* UTEX 1891			No significant similarity	
Volvox rousseletii MI01	vs.	*Volvox dissipatrix* UTEX 2184			No significant similarity	
Volvox rousseletii MI01	vs.	*Chlamydomonas debaryana* CCAP 11/130			No significant similarity	
Volvox rousseletii MI01	vs.	*Vitreochlamys ordinata* Nozaki S-4			No significant similarity	

Comparison of internal transcribed spacer sequences 2 (ITS2) next to the highly conserved 5.8S ribosomal RNA (rRNA) nuclear gene. The list is sorted by expected value (Expect) in ascending order.
